# School-Based Interventions for Increasing Autistic Pupils’ Social Inclusion in Mainstream Schools: A Systematic Review

**DOI:** 10.1007/s40489-024-00429-2

**Published:** 2024-02-13

**Authors:** Yung-Ting Tsou, Lilla Veronika Kovács, Angeliki Louloumari, Lex Stockmann, Els M. A. Blijd-Hoogewys, Alexander Koutamanis, Carolien Rieffe

**Affiliations:** 1 Unit of Developmental and Educational Psychology, Institute of Psychology, Leiden University, Leiden, The Netherlands; 2 Faculty of Science, Vrije Universiteit Amsterdam, Amsterdam, The Netherlands; 3 INTER-PSY, Groningen, The Netherlands; 4 Developmental Psychology, Faculty of Behavioral and Social Sciences, University of Groningen, Groningen, The Netherlands; 5 Faculty of Architecture & the Built Environment, Delft University of Technology, Delft, The Netherlands; 6 Faculty of Electrical Engineering, Mathematics and Computer Science, University of Twente, Enschede, The Netherlands; 7 Department of Psychology and Human Development, Institute of Education, University College London, London, UK

**Keywords:** School-Based Intervention, Social Inclusion, Mainstream Schools, Autism, Systematic Review

## Abstract

School-based interventions for socially including autistic pupils in mainstream schools were systematically reviewed. Included interventions targeted at least one level of the school environment: the autistic children, the peers, the staff, and/or the physical environment, and assessed autistic pupils’ quantity and/or quality of social participation as outcome measures. Findings from 56 studies showed increased accessibility of school activities to autistic pupils, but the reciprocity and friendship between the autistic pupils and the peers were not necessarily improved. Moreover, limited interventions were available for modifying the physical environment. A more holistic strategy that moves the focus from individual children’s social skills to the larger context surrounding children, should be considered for a better inclusion of autistic children in school routine.

School is an important setting for children to meet and socialize with their age-mates and make friends. For many children, school is the only place where they can have peer interactions (Buchanan et al., 2022; Hoffman & Miller, 2020). By interacting with peers, children not only learn the social skills that prepare them for adulthood, such as how to negotiate and collaborate with others (Veiga et al., 2017); but also develop a sense of belonging to the school environment (Allen et al., 2021), which contributes to their psychosocial wellbeing in later life (Palikara et al., 2021; Tian et al, 2016).

However, for many autistic children, socializing in school is no easy task and could even be a major source of stress (Rieffe et al., 2021; Williams et al., 2019). They face many challenges when joining or initiating an interaction with peers (Brewster and Coleyshaw, 2011; Kasari et al., 2012), are often excluded or ignored by allistic (i.e., non-autistic) peers (De Boer & Pijl, 2016; Dean et al., 2014), and the physical environment could simply be too arousing for them to comfortably participate in group activities, such as the playground being too crowded or the hallway being too noisy (Corbett et al., 2014; Mayes et al., 2013; Rieffe et al., 2021; see Bailey and Baker (2020) for a review on the barriers). These challenges reflect the fact that the needs of autistic pupils are not well considered in the organization and design of current school environments, while the allistic preferences for social interaction are promoted. Special considerations are warranted to make schools more welcoming for autistic children. Such considerations are becoming more urgent with the global trend towards inclusive education over the past decade, which means that more and more children with special needs, such as autistic children, are attending mainstream schools.

School-based interventions for the inclusion of autistic pupils generally address one of four levels in a school environment. First, interventions targeting the autistic children (the child level) usually aim at improving the social skills of autistic children, by giving therapist-led training sessions in schools where autistic pupils learn skills to initiate and maintain an interaction based on allistic norms of social interaction (e.g., Dean et al., 2020, Feng et al., 2008, Laushey et al., 2009). Second, interventions that also involve allistic peers (the peer level) often promote autistic pupils’ interactions with their peers by training allistic peer partners or “coaches” to accompany or include autistic children during activities (e.g., Hughes et al., 2013b; Thiemann and Goldstein, 2004), or by forming peer groups with whom autistic pupils regularly meet to discuss school life issues and allistic social rules (e.g., Hart and Banda, 2018; Schaefer et al., 2018). Third, interventions that further involve the school staff (the staff level), e.g., the teachers or the paraprofessionals, usually aim to provide knowledge and training to adults who work directly with autistic children in school, so they acquire the skills to facilitate the interactions between autistic pupils and the peers (e.g., Locke et al., 2019; Kretzmann et al., 2015). Fourth, a small number of intervention programs addresses the physical environment of the classroom or school (the physical environment level), for example, by renovating the school playground with features that encourage autistic children to play together with peers (e.g., Baker et al., 1998), or by changing the seating arrangement to allow allistic peers to have opportunities to be in contact with autistic children (e.g., Chung & Douglas, 2015).

These interventions all tackle a different level of autistic children’s inclusion in schools, but a combined knowledge base is lacking. Previous review studies mostly centered around a single level (e.g., child level: Camargo et al., 2014; Dean & Chang, 2021; peer level: Ezzamel, 2016; Watkins et al., 2015). Although some studies did include multiple levels in their reviews, they primarily focused on the effects of these levels on educational and behavioral functioning, such as academic performance, social skills, and problem behaviors (Lang et al., 2011; Leifler et al., 2021; Watkins et al., 2019). To the best of our knowledge, Sutton et al. (2019) and Whalon et al. (2015) are the only review studies that focused on peer interaction. However, the outcome measures investigated in those reviews either involved only the quantity of peer interactions, i.e., the frequency and duration of social initiations and responses, but not on the quality of these interactions; or included only the social behaviors from the autistic pupils to their peers, rather than the other way around. In other words, thus far the review studies seem to focus on the extent to which interventions allow autistic pupils to “fit in” to allistic peer interactions, and meta-analyses also confirmed moderate-to-strong effect sizes of such interventions that increased autistic pupils’ skills to meet allistic standards of socialization towards their peers (Watkins et al., 2019; Whalon et al., 2015). However, these reviews may not necessarily reflect increased social inclusion of these pupils. This gap in our knowledge prevents us from drawing up a more holistic strategy to address social inclusion of autistic children in mainstream schools.

This current study, in the form of a systematic review, aimed to investigate i) what school-based interventions were available in the evidence base of journals that were designed to enhance autistic pupils’ social inclusion in primary and secondary mainstream schools, ii) at which level of the school environment they targeted at, and iii) the extent to which they were effective. Included interventions should have been designed to target at least one of the four levels of the school environment, i.e., the child-level (the autistic children), the peer-level, the staff-level, and the physical environment level, and adopted a design that allows for an indication of changes in autistic children’s quantity or quality of social participation when an intervention is applied. By synthesizing the knowledge available on this topic, the approaches at each level for socially including autistic children in schools and their effects can be summarized, allowing for a more holistic strategy to be implemented in the school settings.

## Methods

### Literature Search

The Preferred Reporting Items for Systematic Reviews and Meta-Analyses (PRISMA) Checklist was used to guide the review process in this study (Moher et al., 2010). A search was conducted in April 2021 using four electronic databases, i.e., PsycInfo, PubMed, ERIC, and Web of Science, for all peer-reviewed published/in-press literature. Manual search of reference lists of the retrieved studies was conducted afterwards.

Search terms spanning five areas were used in combination with each other: (1) autism (autis* OR pervasive develop* OR Asperger*); (2) children (pupil* OR child* OR adolescen* OR student* OR youth* OR young* OR school age*); (3) school setting (primary school* OR secondary school* OR elementary school* OR high school* OR schoolyard* OR playground*); (4) intervention (interven* OR train* OR adapt* OR program*); (5) social participation (social* OR interaction* OR participation* OR initiation* OR engagement* OR belong* OR bully* OR friend* OR peer*).

### Eligibility Criteria and Study Selection

Given the aim of this review to provide an overview of evidence-based interventions, this review included only studies that have been published in scientific, peer-reviewed journals. The studies should be published in English by the end of April 2021. Gray literature was not included because currently there has not been consensuses regarding how to systematically search for gray studies, include or exclude them in the review process, and evaluate the quality of data from such studies (Martín et al., 2005; Schmucker et al., 2017).

Moreover, a study had to meet the following criteria to be included in this review. First, it involved participants attending primary or secondary schools, and diagnosed with an autism spectrum disorder (ASD; including autism, Asperger syndrome, and pervasive developmental disorder not otherwise specified [PDD-NOS]). Studies were excluded if they were not conducted in primary or secondary schools (e.g., Boyd et al. (2018) only on preschoolers). Given the already wide age range considered in this review, studies were also excluded if they included *only* pupils above 18 years (although no studies were excluded for this reason). When a study included participants with other diagnoses, it was taken into the review process if results specific to autistic pupils were presented (e.g., Schaefer et al., 2018), and excluded if it reported only aggregated data (e.g., Bailey et al., 2021).

Second, the autistic pupils included in the study were in a mainstream, general education setting, which means that these students shared the school context and activities with allistic peers. Therefore, if a study involved only pupils in a self-contained special education class, it was excluded (e.g., Ackerman et al., 2021, Bambara et al., 2016).

Third, the study examined a school-based intervention (e.g., a program, a training session, or an adaptation) implemented at one or more of the four levels of the school environment: the child, the peers, the staff, and/or the physical environment. The intervention aimed to improve the social inclusion of autistic pupils, with a primary outcome measure for social participation with peers in terms of its quantity (e.g., frequency/duration of social interactions, initiations, or responses; number of friends) or quality (e.g., friendship quality, bullying, peer acceptance/rejection, or school belongingness/loneliness).

Fourth, the study should test the effect of the school-based intervention, by adopting a group design (i.e., with an experimental group of pupils who underwent the intervention, compared to a “treatment as usual” control group) or a single subject design (i.e., pupils serving as their own control, whereby their outcomes were examined and compared between baseline and intervention conditions, with at least one measurement to examine each condition).

Fifth, the methodological quality of the study had to be rated as “strong” or “adequate” (Reichow et al., 2008; see below for more details). Studies rated as “weak” were excluded.

**Fig. 1:**
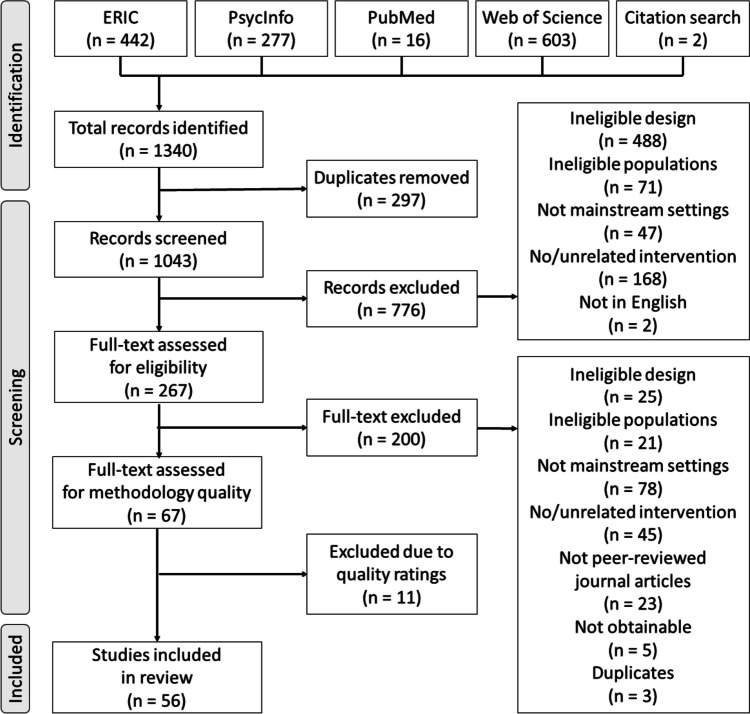
PRISMA flow chart of the selection process

The selection process involved two stages: first, the duplicates were excluded and the titles and abstracts were screened; second, the full texts were reviewed for eligibility. The screening and eligibility check were conducted by two individual coders (the second and third authors of this study). In both stages and throughout the review process, all studies were coded by the two coders individually, and disagreements were discussed between the two coders and a third tiebreaker (the first author) until reaching 100% agreement in biweekly project meetings. A training session took place before each stage for the discussion about the criteria and their definitions, during which five articles were coded iteratively until 100% agreement was reached. With this set-up, 96% and 99% agreement was respectively achieved in the two stages regarding which studies to exclude. The complete review process is presented with a PRISMA flow chart in Fig. 1.

### Methodological Quality

The methodological quality of the reviewed studies was assessed using the evaluation protocol for autism research proposed by Reichow et al. (2008). For group research, there are six primary quality indicators (i.e., participant characteristics, independent variables, dependent variables, comparison condition, link between research question and data analysis, and use of statistical tests) and eight secondary quality indicators (i.e., random assignment, interobserver agreement, blind raters, fidelity, attrition, generalization and/or maintenance, effect size, and social validity). A study was considered “strong” if it met all the primary indicators and at least four secondary indicators. When a study met at least four primary indicators and at least two secondary indicators, it was rated as “adequate.” Other studies were considered “weak.”

Single subject research can be assessed with six primary indicators (i.e., participant characteristics, independent variables, dependent variables, baseline condition, visual analysis, and experimental control), and six secondary indicators (i.e., interobserver agreement, kappa, fidelity, blind raters, generalization and/or maintenance, and social validity). When a study met all the primary indicators and at least three secondary indicators, it was deemed “strong.” An “adequate” study met at least four primary indicators and at least two secondary indicators. Other studies were rated as “weak.”

As mentioned, two coders coded all studies based on the provided protocol. After the first round of quality assessment, however, 36% of the studies were deemed “weak” by one coder and “adequate” by the other. Therefore, the third tie-breaker joined the second round of quality assessment. The protocol was reviewed again among the coders to resolve any concerns, and all the studies were discussed one-by-one until 100% agreement was achieved in the biweekly project meetings.

### Data Extraction

The two independent coders each extracted data from all the eligible studies. Study characteristics were extracted through open-ended questions and/or forced choice questions. For open-ended questions, agreement was considered achieved when the coders selected the same range of information from the studies. For forced choice questions, agreement was reached when the same option was chosen. Before data extraction, a training session was organized where five articles were coded for practice and 100% agreement must be met. The coded data was discussed one-by-one in regular project meetings. Disagreements occurred in about 14% of the studies, due to doubts about the setting the autistic pupils were in, which measures could be seen as reflecting social participation, and the outcomes being compared. Also when extracting effect sizes, disagreements occurred in 25% of the studies. These disagreements were discussed with the third tie-breaker in regular project meetings until agreement was achieved among all coders.

The data extracted included: i) study characteristics; ii) participants characteristics; iii) setting; iv) measures; v) intervention characteristics; and vi) outcomes. See Table 1 for an overview of the characteristics that the two coders extracted. These characteristics were chosen primarily based on the research question of this study regarding the different levels of the school environment and the effects we aimed to examine. Moreover, they were chosen to meet the requirements of the methodological quality evaluation protocol of Reichow et al. (2008). Previous review studies (e.g., Sutton et al., 2019; Whalon et al., 2015) were also taken into account to finalize the list of characteristics for the extraction.

**Table 1:** Overview of characteristics extracted for each reviewed study

	Type of question that coders addressed during data extraction	
Type of data extracted	Open-ended question	Forced-choice question
i. Study characteristics	Country of origin; Inclusion/exclusion criteria; Recruitment procedure; Allocation	Study design (group or single-subject);
ii. Participants characteristics	Number of participants (all/autistic); Age (mean; range in years) or grade; Gender distribution (female: male); ASD diagnosis (name diagnosis, *n*); Additional diagnoses/disabilities (name diagnosis, *n*)	-
iii. Setting	-	School setting (primary or secondary); Inclusion method adopted by the school (individual inclusion, group inclusion, or special classes)
iv. Measures	Dependent variables (one entry per variable); Measurement instruments; Number and length of measurements (*n* baseline; *n* during intervention; *n* after intervention, *n* follow-up; others)	-
v. Intervention characteristics	Intervention approach; Number of cycles; Timeframe; Co-interventions	Targeted level of school environment (child, peers, staff, or physical environment); Interventionist/trainer (researcher or teacher/school staff);
vi. Outcomes	Comparison outcomes; Statistical techniques; Effect size	-

## Results

### Study Characteristics

A total of 56 studies met the criteria for the current review (see Table 2 for an overview, and Appendix Table 4 for a complete list of included studies and corresponding outcomes). Among them, 47 (84%) employed a single subject design, while 9 (16%) adopted a group design.

**Table 2:** Participant characteristics

Type of studies	N reviewed studies (%)	N autistic participants (%)
All reviewed studies	56 (100%)	981 (100%)
Including girls	24 (43%)	138 (14%)
Autism diagnosis		
Autism	31 (55%)	332 (34%)
ASD	26 (46%)	611 (62%)
Asperger’s syndrome	10 (18%)	19 (2%)
No diagnosis but meeting ADOS criteria	2 (4%)	5 (1%)
Not on the spectrum based on ADOS	1 (2%)	14 (1%)
Additional diagnoses		
Intellectual disability	8 (14%)	28 (3%)
Speech or language impairment	5 (9%)	7 (1%)
ADHD	6 (11%)	7 (1%)
Anxiety disorders	2 (4%)	2 (0.2%)
Oppositional defiant disorder	1 (2%)	1 (0.1%)
Down syndrome	1 (2%)	1 (0.1%)
Seizure disorder	1 (2%)	1 (0.1%)
Hearing loss	1 (2%)	1 (0.1%)
Visual disability	1 (2%)	1 (0.1%)
Specific learning disability	1 (2%)	1 (0.1%)
School setting		
Primary schools^a^	35 (63%)	735 (75%)
Secondary schools	18 (32%)	197 (20%)
Primary and secondary schools	3 (5%)	49 (5%)
Country of origin		
Canada	1 (2%)	3 (0.3%)
Ireland	1 (2%)	30 (3%)
Spain	1 (2%)	1 (0.1%)
Taiwan	1 (2%)	1 (0.1%)
United Kingdom	1 (2%)	1 (0.1%)
United States	51 (91%)	945 (96%)

*Note*. ADHD = attention deficit hyperactivity disorder; ADOS = Autism Diagnostic Observation Scale; ASD = autism spectrum disorder

^a^ Two studies also include kindergarten children

Almost all studies were conducted on Western samples, in Canada (1), Ireland (1), Spain (1), United Kingdom (1), and United States (51). Only one study was on a non-Western sample, in Taiwan (0.1% of all the autistic children involved in this review; Feng et al., 2008).

Twenty (36%) studies received a “strong” methodological quality rating, while 36 (64%) were rated as “adequate” (see Appendix Table 5 and 6). Overall, the studies eligible for this review described the participants, the intervention, and the outcome measures in detail, conducted data analyses that fit the research questions, and provided information about generalization or maintenance of the targeted behaviors, thus making replications possible.

**Table 3:** Summary of outcome measures and corresponding outcomes

Outcome measure		Child level			Peer level			Staff level	Physical environment level	All levels	N per measure type
		Child only	Joined by peers	Joined by staff	Peers only	Researcher facilitator	Staff facilitator				
Observation	Initiation	** + (1/1)**; = (2/0)	** + (2/0);** = (2/0)		** + (4/0);** = (3/0)	** + (4/0)**	** + (5/2);** = (1/0)	** + (1/0)**	** + (2/0);** = (1/0)	** + (1/0)**	54
	Response	** + (1/0);** = (2/1)	** + (3/0);** = (1/0)		** + (1/0)**	** + (3/0)**	** + (5/0)**				
	Peer initiation				** + (1/0)**	** + (1/0)**	** + (2/1)**			** + (1/0)**	
	Peer response	= (1/0)			** + (1/0)**	** + (2/0)**	** + (2/0)**				
	Interaction	** + (2/1);** = (0/1)	** + (1/0)**		** + (6/0)**	** + (2/0)**	** + (7/2)**	** + (2/0)**	** + (1/0)**	** + (1/0)**	
	Engagement; joint activity	** + (3/0);** = (2/0)	** + (2/0)**	** + (1/0)**	** + (3/0)**	** + (1/0)**	** + (2/0)**	** + (2/2);** = (0/1)	** + (1/0)**		
Peer nomination/ rating	Peer preference^#^	= (1/2)^bcd^	** + (2/0)**^**cd**^ ; = (1/0)^b^		** + (0/1)**^**bd**^; = (0/1)^ac^	** + (2/0)**^**a**^ ; = (1/0)^bd^	= (1/0)^a^	** + (2/0)**^**d**^ ; = (2/0)^b^			9
	Self-perceived connections	= (1/1)	** + (1/0)**		= (0/1)	= (1/0)		** + (0/1)**			
Parent report	Social participation					** + (1/0)**	= (0/1)				2
Teacher report	Social participation		** + (1/0)**				** + (0/2)**				3
Self report	Victimized					**- (1/0)**					1
N per type of intervention		12	5	1	12	6	12	7	4	1	

*Note*. The “ + ” mark indicates an increase in the outcome measure (for single-subject design) or a higher level of the outcome measure in the experimental group compared to the control group (for group design). The “-” mark indicates a decrease in or a lower level of the outcome measure. The “ = ” mark indicates no effect of intervention on the outcome measure. Numbers in the parentheses denote the number of studies with a single-subject/group design that reported a specific outcome. When a cell is empty, it shows an absence of such a measure in the reviewed studies

^#^ Peer preference involves all the outcome measures that include the nominations or ratings given by peers. ^a^ Peer acceptance scores. ^b^ Friendship nominations given by peers. ^c^ Reciprocated friendship nominations. ^d^ Social network centrality

### Participant Characteristics and Settings

A total of 981 autistic children were involved in this review (see Table 2). Among these, 138 (14%) were girls, yet four studies did not specify the sex of the participating autistic children. The sample size of autistic children was variable, from 1 to 197 autistic pupils.

Participants were reported as diagnosed with autism (*n* = 332), Asperger’s syndrome (*n* = 19), or ASD (*n* = 611). Five children did not have an autism diagnosis but met the criteria when assessed with the Autism Diagnostic Observation Scale (ADOS). Fourteen children who had an autism diagnosis when recruited were no longer on the spectrum during the intervention according to ADOS. Also, 50 children were reported to have additional diagnoses, including intellectual disability (*n* = 28), speech or language impairment (*n* = 7), attention deficit hyperactivity disorder (ADHD; *n* = 7), anxiety disorders (*n* = 2), oppositional defiant disorder (*n* = 1), Down syndrome (*n* = 1), seizure disorder (*n* = 1), hearing loss (*n* = 1), visual disability (*n* = 1), and specific learning disability (*n* = 1). In most studies (*n* = 37; 66%), the inclusion criteria only entailed an autism diagnosis, without specifying functionality or cognitive levels.

Thirty-five studies were conducted among primary-school students, although two of these studies also included kindergarteners (*n* = 1 [17% of the sample] in Vincent et al. (2018); and number unknown in Morgan et al. (2018)). Eighteen studies involved only secondary-school students, and three studies included children from both primary and secondary schools (Brock et al., 2018; Hanley-Hochdorfer et al., 2010; Kamps et al., 2002). In addition, two studies reported aggregated results of a sample that also included autistic children from self-contained special classes besides the autistic pupils from mainstream classes (*n* = 1 [25% of the sample] in Kamps et al. (2014); *n* = 117 [59% of the sample] in Morgan et al. (2018)).

### Outcome Measures

As Table 3 shows, observations were the most used method (*n* = 54; 96%) for measuring autistic children’s social inclusion in school. Although the instruments used were wide-ranging, the dependent variables can be categorized into four types: initiations, responses, interactions, and engagement. First, a total of 35 studies (63%) examined the frequency and/or quality (valence, appropriateness, verbal or not, and prompted or not) of social initiations, from the target autistic children to their peers (*n* = 29), and/or from the peers to the autistic children (*n* = 6). Second, 22 studies (39%) examined the frequency and/or quality of the social responses, from the autistic children to their peers (*n* = 17), and vice versa (*n* = 5). Third, 26 studies (46%) looked at the back-and-forth nature of the observed interactions (e.g., counting both initiations and responses; the presence of turn-taking; the presence of reciprocal exchanges), and among them, two studies also investigated the time when the autistic children were alone. Lastly, 22 studies (39%) checked if the autistic children were engaged in an activity, whether jointly with peers (*n* = 21), solitarily (*n* = 3), or near other peers but doing a separate activity (*n* = 1).

Besides observations, peer nominations were conducted in nine studies (16%), where all participating students (autistic and allistic) were asked to nominate several friends or playmates, or to rate a certain aspect about their interaction with a given peer (e.g., whether they like to play with the peer). Through these nominations and ratings, autistic pupils’ sociometric status was measured. Some studies also collected self-report questionnaires from autistic children themselves (*n* = 1; 2%), or informant-report questionnaires from their parents (*n* = 2; 4%) or teachers (*n* = 3; 5%), to examine these children’s social participation in schools or outside the intervention.

### Statistical Analyses

Among the 47 studies where a single-subject design was utilized, the majority (*n* = 43; 91%) conducted visual inspection/analysis and reported the descriptives. In these studies, levels, trend, and variability of data were inspected, and the immediacy of intervention effect, overlap between phases, and consistency of the patterns were examined. Twelve of these studies reported effect sizes, using Tau or Tau-U (*n* = 4; Kamps et al., 2014; Levy & Dunsmuir, 2020; Mason et al., 2014; Sabey et al., 2020); non-overlap of all pairs (NAP) (*n* = 4; Block et al., 2015; Radley et al., 2014, 2017; Rodríguez-Medina et al., 2016); percentage of non-overlapping data (PND; PNOD) (*n* = 2; Carter et al., 2017; Hanley-Hochdorfer et al., 2010); pairwise data overlap (PDO) (*n* = 1; Laushey et al., 2009); improvement rate difference (IRD) (*n* = 1; Brain & Mirenda, 2019). Besides visual inspection, one study also used a nonparametric Wilcoxon matched pairs test and reported *r* statistics for the comparison of pre-post friendship nominations (Rodríguez-Medina et al., 2016); two studies also conducted analyses of variance (ANOVA) (Frederickson et al., 2005; Kamps et al., 2002), where one of them (Frederickson et al., 2005) reported eta square values for effect sizes. Three studies with a larger sample size used (generalized) linear mixed models to model the changes over the different assessments (*N* of autistic pupils = 31 in Locke et al. (2019); *N* = 32 in Dean et al. (2020); *N* = 137 in Kasari et al. (2016)). Two studies among them specified the effect sizes with Cohen’s *f* (Dean et al., 2020; Locke et al., 2019).

Among the nine studies that adopted a group design, six studies used mainly multi-level modeling techniques (linear mixed models (LMM); hierarchical linear models (HLM)) to analyze the effects, while one study applied a generalized approach with truncated Gaussian models (Shih et al., 2019). In the remaining studies, one study used the analyses of covariance (ANCOVA) (Golzari et al., 2015), while the other used ANCOVA for comparing between groups in peer nominations and HLMs for modeling playground observation data (Kasari et al., 2012). The effect sizes were reported in all of these studies, using Cohen’s *d* (*n* = 7; Asmus et al., 2017; Brock et al., 2018; Carter et al., 2016; Kasari et al., 2012; Kretzmann et al., 2015; Lopata et al., 2019; Morgan et al., 2018); Cohen’s *f* (*n* = 1; Shih et al., 2019); eta square (*n* = 1; Golzari et al., 2015).

### Type of Interventions and Outcomes

Twelve (21%) studies applied the intervention at child level; nine (16%) at peer level; seven (13%) at staff level; and four (7%) at physical environment level. The remaining 24 (43%) studies implemented the intervention at multiple levels of the school environment: four targeting at both the child and the peers, and one at both the child and the staff; 18 targeting at the peers, but also adding an adult facilitator who was either someone from the research team (*n* = 6) or a school staff who received training (*n* = 12); and one covering all of the four levels (Chung & Douglas, 2015).

#### Child Level

The studies that examined interventions at child level focused on implementing a training program, led by a therapist or the researcher separately from the allistic peers, for improving autistic children’s social skills (see Appendix Table 4). A commonly applied program was the Social Stories (Gray, 2010; Gray & Garand, 1993), which provides specific instructions for behavioral responses, such as how to appropriately join in and maintain an interaction within a defined context, via visual supports and text (Delano & Snell, 2006; Golzari et al., 2015; Hanley-Hochdorfer et al., 2010; Sansosti and Powell-Smith, 2006; all the four studies explicitly mentioned that the guidelines of Carol Gray for the Social Stories were followed). One study further provided teaching scripts to special education teachers in the school for teaching social skills through stories (Bock, 2007). Similarly, programs like the Concept Mastery Routine focus on defining a social concept, e.g., appropriate social initiations, with autistic pupils using visual diagrams (Laushey et al., 2009).

Some studies also included a small group of allistic peers in social skill training. These peers (trained or untrained) jointly acted as a collaborative reading partner after the training sessions (Reutebuch et al., 2015), or as models or role-playing partners when autistic pupils were taught or practiced social skills like joint attention, turn-taking, responding to questions, and maintaining conversations (e.g., the Superhero program, such as in Radley et al. (2014) and Block et al. (2015)). One study involved peers indirectly: autistic children had to nominate a peer to play with and was prompted to do so during recess (Kasari et al., 2016).

As Table 3 shows, following such interventions targeting at social skills, autistic children spent more time interacting with peers, engaged more often in joint activities with peers, and made initiations and responses to peers in a manner that more often met the researchers’ definition of “appropriateness.” Teachers also reported a higher level of social participation in autistic pupils after the intervention. However, these interventions did not seem to improve peers’ responses to the target autistic children. Whether with a group or single-subject design, no changes were observed in peers’ responses to the target (whether the responses were positive, negative, or absent). Similarly, no changes were noted in peer rejection or friendship nominations, as reported from peers.

#### Peer Level

The interventions at peer level usually involved trainings to teach allistic peers how to interact with an autistic classmate, and these peers were assigned as partners or life coaches to accompany autistic pupils during recess or in a certain school activity (e.g., Brain & Mirenda, 2019; Carter et al., 2016; Thiemann and Goldstein, 2004; see Appendix Table 4). Another common peer-mediated intervention is forming peer networks, where a group of allistic peers were trained beforehand, and teamed up with autistic pupils outside the regular school hours to have discussions on different issues related to social situations and interactions or on shared interests (e.g., Haring and Breen, 1992; Hochman et al., 2015; Kasari et al., 2016). These peer-network interventions were often in combination with an adult facilitator, either the researcher or a teacher in the school. For studies that included a school staff member as facilitator (e.g., Asmus et al., 2017; Carter et al., 2016; Rodríguez-Medina et al., 2016), trainings were given to staff beforehand to demonstrate strategies for facilitating discussions and interactions between autistic children and the allistic peers. In one study, all the allistic peers in a class participated in an adult-facilitated session, in which they discussed friendship and the focal autistic child’s strengths and difficulties (without the focal child’s presence), and afterwards the allistic peers who volunteered were included in the peer network (Frederickson et al., 2005).

As presented in Table 3, in interventions with trained allistic peers as partners who accompanied the autistic children in school activities or during recess, it was found that the reciprocity in peer interactions increased: there were more responses from peers to the target autistic child; more verbal exchanges and turn-taking between the autistic child and the peers; and the interactions were maintained for a longer time. The results of peer nominations also showed that autistic children received more friendship nominations from the peers and were in a more central position in a social group. Nevertheless, there were no clear effects on autistic children’s initiations to peers, and no changes in the friendship nominations given by autistic children. There were also no differences in peer acceptance when trained and untrained peers were both included in the investigation.

When peer-network meetings were held, the studies that included the researcher as the facilitator and those that included a schoolteacher as the facilitator produced similar results. They showed that, outside the peer network sessions, autistic children spent more time in peer interactions and engaging in joint activities with peers; there were more initiations and responses to and from the peers; and the peer acceptance increased while peer rejection and victimization decreased. Yet, although the autistic children engaged more often with peers, they did not get more friendship nominations from the peers, nor nominated more friends themselves. Furthermore, while teacher reports showed increased social contacts and gaining new friends, parents reported no changes in social contacts and friends.

#### Staff Level

The interventions at staff level all involved training programs for school teachers or paraprofessionals, aimed at helping school staff identify needs of autistic children and promote child-led activities (see Appendix Table 4). Among these, three studies made use of the Remaking Recess program that trained teachers or paraprofessionals to facilitate peer interactions specifically during school recess on the playgrounds, while the other programs were applied to general school settings (Kretzman et al., 2015; Locke et al., 2019; Shih et al., 2019). Also, three studies taught paraprofessionals to include the Pivotal Response Treatment procedures, a naturalistic behavioral methodology, in supporting autistic children’s peer interactions in school activities (Feldman & Matos, 2013; Pierce & Schreibman, 1997; Robinson, 2011).

Interventions that provided staff training had effects on autistic children’s engagement in joint activities with peers, their initiations to peers, and reciprocity in their interactions (see Table 3). Autistic children were also reported to nominate more friends and to be in a more central position in social groups according to peer reports. However, no differences were observed in the friendship nominations they received from peers, regardless of the study design.

#### Physical Environment Level

Only four studies targeted specifically at physical environment (see Table 3 and Appendix Table 4). Among them, two studies by the same research group launched social clubs in schools based on the autistic pupils’ preferred interest (e.g., a movie or a frisbee club; Koegel et al., 2012, 2013). Similarly, one study turned the special interests of autistic children into playground games (e.g., interest in geography was incorporated into a tag game on a giant map outlined on the playground; Baker et al., 1998). The remaining one study provided structured activities that motivate peer interactions (e.g., relay races, board games, and scavenger hunts) on the playgrounds (Vincent et al., 2018). These activities were open to all students in the schools or on the playgrounds, although in two studies, children’s social interactions were facilitated by an adult (Baker et al., 1998; Vincent et al., 2018). After the interventions, it was observed that autistic pupils spent longer time engaging in joint activities with peers and made more initiations to their peers. However, no studies examined peers’ responses, and only one study followed up to see if the effect maintained over time.

There was also one study that applied a combined strategy (Chung & Douglas, 2015), including all four levels of the school environment: offering speech generating devices, inviting peer partners, training paraprofessional facilitators, and rearranging the class seating to allow the target autistic students to sit with their allistic peer partners. The study however only examined the aggregated effects, and found improvements in the reciprocity during peer interactions, with more initiations by both the target autistic child and the peers.

## Discussion

School is the place where many children spend most of their waking hours, acquiring new academic and physical skills, learning social conventions, hanging out with peers, and making friends. Providing a socially inclusive school environment for all children is therefore a necessity. However, this goal appears quite difficult to attain for many schools, partly due to the gap in our knowledge regarding which solutions are available and which ones work for whom.

This systematic review showed that interventions at different levels of the school environment can effectively increase autistic pupils’ interactions with their peers, yet it remains unclear whether these school-based interventions led to better social inclusion for these children. Below we discuss the reviewed outcomes and their implications for practice and future research.

### Levels of Social Inclusion

A recent editorial article by Weaver and colleagues (Weaver et al., 2021) proposed the “community engagement continuum” for defining the extent to which an autistic individual is socially included in a community. This framework includes four layers: (1) *tolerance*, where an individual prepares tools themselves to be physically present in an environment and tolerates the unmodified environment; (2) *accessibility*, where an individual is given supports or accommodations to physically access an environment, but no changes are made for supporting social participation; (3) *integration*, where an individual is given support and opportunities for both physical engagement and meaningful social participation; and (4) *inclusion*, where an environment (e.g., social, cultural, or physical environment) is modified to allow all individuals to belong and contribute meaningfully.

Following this line of thought, interventions at the child level seem to relate to *accessibility*, as these programs do not create opportunities for social participation; instead, autistic children are taught social skills, with which they use to create those opportunities themselves. Echoing this definition of accessibility, the current review showed that autistic children did have an increased presence in joint activities with peers, and made more attempts to initiate an interaction or respond to peers, after receiving the child-specific social skill training in school. However, peers’ responses to the autistic children did not change. In fact, even the proportion of “no response” from peers stayed unchanged (Sabey et al., 2020), showing that autistic children were still ignored by their peers – a form of implicit aggression – regardless of their improved social skills.

As to the interventions at the levels of peers and staff, *integration* was achieved to a certain degree. Through training, (some of) the allistic peers and staff improved their interaction/facilitation skills and the understanding of the difficulties surrounding autism, which led to more reciprocity in the interactions between the autistic children and their peers. However, having more peer interactions does not necessarily mean making more friends. Whilst an adult facilitator could steer more social initiations and responses among children, the friendship nominations received by the autistic children appeared unaffected after the intervention, showing that these children were still not seen as a friend by their peers (e.g., Kasari et al., 2016). Moreover, although having the company of peer partners led to more peer responses and more friendship nominations from the peers, the autistic children seem to adopt a passive position in such peer-mediated interactions, which resulted in producing no effect on their initiations to peers nor on the friendship nominations made by themselves (e.g., Carter et al., 2016; Kasari et al., 2012). Also, the positive effects derived from these programs may not extend to the untrained peers and staff.

In theory, interventions applied at the level of physical environment could be an approach for achieving *inclusion*. The modifications made to the physical environment for fitting individual needs bring the message that individual differences are valued and respected, and that is the starting point for an autistic child to feel belonged in school. In the current review, positive effects were shown in three school-based interventions of this sort, including providing playground games and materials that encourage social interactions (Vincent et al., 2018), designing new playground activities based on autistic children’s preferred interests (Baker et al., 1998), and forming school social clubs based on autistic children’s preferred interests (Koegel et al., 2013). However, given the small body of literature and the fact that peers’ responses were not recorded in these studies, it is hard to confirm from the current review the extent to which autistic children may benefit from such approaches. This presents an urgency to understand the changes in peers’ behaviors towards autistic children after adaptations are made to the physical environment in schools.

### Practical Implications, Limitations, and Future Research

As one of the first systematic review that addresses social inclusion of autistic children at different levels of mainstream school environment, this study provides an overview of school-based, methodologically sound interventions currently available to educators and researchers. It carries several implications for practice and future research, while some limitations should also be considered.

In this review, child-specific and peer-mediated interventions were the most common approaches, yet it is worth noting that such interventions mostly took place outside the regular school routine. For example, child-specific sessions and peer network meetings were held in a separate room from children’s usual classroom, and peer partners received the training outside the curriculum. Such an outcome shows that social inclusion remains an extra layer of school education and may reflect the harsh reality that faces mainstream school educators with a dearth of means for increasing social inclusion among children within the school routine.

However, when an intervention for social inclusion is considered an addition to school routine and focusing specifically on a child’s social skills, stigmatization is likely to occur (Turnock et al., 2022); and worse, when unsuccessful the child might feel he or she failed, most likely further damaging their self-esteem, their position in the group, and their sense of belonging (Rieffe et al., 2018). The prevalence of such a child-specific point of view among the reviewed studies should therefore be taken with caution. The fact that most of the reviewed studies measured only autistic children’s interactions towards peers, and provided limited information in the other way around, may also reflect an underlying child-specific perspective in the choice of measures in many studies. There was also a lack of differentiations in regard to autistic pupils’ motivation towards socialization and these children’s perception of “meaningful” participation. It should be noted that having a higher quantity of social interactions, such as making more initiations and responses, or having more “appropriate” interactions, does not indicate that these interactions are also of higher quality. Individual differences in what makes an interaction enjoyable, and the effects of the surrounding social and physical environment, must be accounted for when evaluating an intervention.

Therefore, future researchers as well as schools and teachers are encouraged to switch focus from “fixing” autistic pupils, to addressing the school environment that surrounds autistic pupils, including the awareness of diversity and equity among peers and staff, and the design of the physical environment such as providing places to seek quiet and more green spaces (e.g., Rieffe et al., 2021; also see Martin (2016) for an overview of recommendations for physical environment design for autistic individuals). To this end, two aspects require special attention.

First, special attention should be paid to the measures for evaluating the interventions across the different levels of school environment, including the experiences of the autistic pupils. Currently, data is primarily from observations, presumably by allistic observers. Future studies should consider including other methods, such as questionnaires by autistic pupils and other relevant informants (e.g., peers, caregivers, or teachers) to better account for autistic pupils’ perspectives and experiences, and to confirm that the effects are maintained outside the observed contexts. Moreover, recent research showed that sensing technologies may be used to assess social dynamics at both group and individual levels, and the interaction between pupils and the built structures (Eichengreen et al., 2024; Nasri et al., 2022).

Second, tools should be developed to support schools and teachers to create a welcoming setting where diverse needs and desires are respected and valued. Notably, the current review focused on primary and secondary schools that provide mainstream education, where autistic children shared (most of) their school time with allistic peers. Practices in special education schools were thus not considered in this review. However, these practices might be insightful to the design of interventions that fit the needs and experiences of autistic children also in other school settings. For example, Yuill and colleagues (Yuill et al., 2007) examined the effect of a new playground that was designed for autistic pupils, in a special-education primary school. This new playground provided a clear circuit between activities (e.g., the slide curved to the direction where the next activity starts) to encourage playful movements and interactions naturally and structurally. It also had observation points where autistic children could observe others’ play without needing to interact, or recover from sensory overarousal triggered by the surroundings, which is often experienced by autistic individuals.

Notably, this review included both group design and single-subject design, in the attempt to cover all published studies that evaluated the effect of an intervention. Yet, it should be taken into account that the majority (84%) of the included studies were of a single-subject design and thus mostly with a small sample size. Also, single-subject designs are prone to internal validity risks if the conventions are not adhered to and stability within conditions cannot be established (Petursdottir & Carr, 2018). In this review, we mitigated the effects of these risks by evaluating the studies’ methodological quality and synthesizing only the results from those with an adequate-to-strong quality rating. However, their potential effects should still be taken with caution.

Furthermore, this review did not include gray (unpublished) literature, due to the lack of guidelines for systematically searching, selecting, and evaluating such studies (Martín et al., 2005; Schmucker et al., 2017). Although this decision was based on our aim to provide an overview of evidence-based interventions, we could not rule out the possibility that publication biases might be present in our synthesis as a result (Tincani & Travers, 2019). Thus, it should be noted that some potentially effective interventions might be omitted in this review because they were not published in peer-reviewed journals nor in English.

## Conclusion

The current synthesis of results shows that the school-based interventions currently available in the literature can improve the *accessibility* of school activities, helping autistic pupils gain skills to approach the peers; and can enhance the *integration* of autistic pupils in schools, through the support of trained peers and/or school staff. Yet, a more holistic strategy that interconnects the different levels of the school environment, moving the focus from individual children’s social skills to the modification of the larger context, is required to ensure the *inclusion* of autistic children in schools, where they can meaningfully contribute. It is thus recommended that future studies attend the social, cultural, and physical environments that surround autistic children, and the expectations and experiences of these children concerning social participation. The paucity of information regarding these aspects in the current literature prevents us from drawing conclusions about autistic children’s social inclusion in schools. To fill this gap, new methodologies for measuring children’s interactions with the environments (e.g., a multidisciplinary approach combined with sensor technology; e.g., Andersen et al., 2019; Veiga et al., 2017), and the use of measures that can reflect children’s own views (e.g., with self-reports and focus group interviews), are needed. Such considerations will improve social inclusion not only for a specific population, but for all children, who have different capacities and wishes.

## Data Availability

The dataset and associated information used in the current study will be shared publicly on the Leiden University archiving platform DataverseNL (10.34894/VNKIFG) within one month after publication.

## Appendix

Tables 4, 5 and 6

**Table 4:** Overview of the reviewed studies at each level of school environment (*N* = 56)

Study	N (All/ASD)	N ASD girls	Age (years)	Setting	Intervention	Outcome measure	Analysis method	Results (Effect size)
**Child Level**								
Dean et al., 2020 (US)	30/30	4:26	15	2	Skill training: say “hi”, walk together to bus, socialize with members outside of group sessions (SKILL)	Obs, engaged solo Obs, engaged joint	LMM, visual inspection	**Pr > Po (f**^**2**^** = .08) Pr < Po (f**^**2**^** = .06)**
						Obs, engaged parallel		Pr = Po (f^2^ < .01)
Delano & Snell, 2006 (US)	1/1	0:1	9	1	SOCIAL STORIES	Obs, engaged joint proper Obs, N INIT / RESP	visual inspection, descriptives	Pr = Po Pr = Po
Feng et al., 2008 (TW)	1/1	0:1	11	1	Theory of Mind & social skill training: greet, express emotions/needs	Obs, N INTER Obs, N INTER proper	visual inspection, descriptives	**Pr < Po** **Pr < Po**
Hanley-Hochdorfer et al., 2010 (US)	4/4	1:3	6–12	1 + 2	SOCIAL STORIES	Obs, N INIT verbal Obs, N RESP	visual inspection, descriptives	Pr = Po (PND = 1–38%) Pr = Po (PND = 11–36%)
Hartzell et al., 2015 (US)	3 SN/2	2:0	7	1	Skill training: talk to peers, eye contact, body language, maintain topic	Obs, engaged joint	visual inspection, descriptives	**Pr < Po**
Kasari et al., 2016 (US)	57/57	9/48	8	1	Skill training: greet, nonverbal talk, humor, coping, friendship tips (SKILL)	Obs, engaged joint	GLMM	**Pr < Po**
						PN, centrality PN, outdegree PN, indegree		Pr = Po Pr = Po Pr = Po
Laushey et al., 2009 (US)	4/4	0:4	1-4th grade	1	Skill training through concept diagrams (CONCEPT MASTER ROUTINE)	Obs, N RESP to question Obs, N INIT	visual inspection, descriptives	**Pr < Po (PDO = .67–1)** **Pr < Po (PDO = 1)**
Sabey et al., 2020 (US)	3/3	1:2	7–11	1	Skills training: start a conversation, join a game, ask others to join a game	Obs, N INTER proper verbal	visual inspection, descriptives	**Pr < Po (Tau-U = .91)**
						Obs, N P-RESP pos/neg/none		Pr = Po
Sansosti & Powell-Smith, 2006 (US)	1/1	0:1	10	1	SOCIAL STORIES	Obs, join social activities	visual inspection, descriptives	Pr = Po
Golzari et al., 2015 (IR)	30/30	0:30	6–12	1	SOCIAL STORIES	Obs, N INIT Obs, dur INTER	ANCOVA	**C < E (ƞ**^**2**^** = .20)** **C < E (ƞ**^**2**^** = .23)**
						Obs, N RESP		C = E (ƞ^2^ = .12)
Kasari et al., 2012 (US)	15/15	0:15	8; 6–11	1	Skill training: playground games, enter game/conversation (CHILD)	PN, centrality	HLM, ANCOVA	C = E (d = 0.36)
						PN, indegrees PN, outdegrees PN, rejection PN, bidegree Obs, engaged solo/joint		C = E C = E C = E C = E C = E
Lopata et al., 2019 (US)	103/103	9:94	9	1	Skill training: social skills, mind reading, cooperative group activities; parent training (SCHOOLMAX)	Obs, dur INTER pos	LMM	C = E (d = .08)
***Child***** + *****Peer***								
Block et al., 2015 (US)	4/4	1:3	8–10	1	Skill training: join activity, nonverbal communication, turn-taking, maintain topic (SUPERHEROES)	Obs, N RESP	visual inspection, descriptives	**Pr < Po (NAP = .83–1)**
						Obs, N INIT		Pr = Po (NAP = .78–1)
Radley et al., 2014 (US)	4/4	0:4	8–10	1	SUPERHEROES	Obs, engaged level Obs, N INIT pos Obs, RESP pos PN, outdegree PN, centrality	visual inspection, descriptives	**Pr < Po (NAP = .93–1)** **Pr < Po (NAP = .78–1)** **Pr < Po (NAP = .76-.92)** **Pr < Po** **Pr < Po**
						Obs, N INIT neg Obs, RESP neg PN, indegree		Pr = Po (NAP = .47-.56) Pr = Po (NAP = .23-.57) Pr = Po
Radley et al., 2017 (US)	5/5	0:5	9	1	SUPERHEROES	Obs, engaged joint TR, social participation PN, friends	visual inspection, descriptives	**Pr < Po (NAP = .95)** **Pr < Po** **Pr < Po**
Reutebuch et al., 2015 (US)	3/3	1:2	16	2	Social skill training & reading with a peer	Obs, N INIT Obs, N RESP	visual inspection, descriptives	**Pr < Po** **Pr < Po**
Rosenberg et al., 2015 (US)	3/3	1:2	7	1	Target child identifies a peer to play with and prompted to do so (SAY-DO)	Obs, N INTER verbal	visual inspection, descriptives	**Pr < Po**
***Child***** + *****Staff***								
Bock, 2007 (US)	4/4	0:4	9–10	1	Stories & teaching scripts (SODA: Stop, Observe, Deliberate, Act)	Obs, join social activities	visual inspection, descriptives	**Pr < Po**
**Peer Level**								
Brain & Mirenda, 2019 (CA)	3/3	0:3	12	2	Peers trained as partners/coaches	Obs, engaged joint Obs, N INIT verbal	visual inspection, descriptives	**Pr < Po (IRD = .95)** **Pr < Po (IRD = .95)**
Carter et al., 2017 (US)	3/3	0:3	18	2	Peer trained as partners/coaches	Obs, dur INTER Obs, reciprocity Obs, N INIT	visual inspection, descriptives	**Pr < Po (PND = 83–100%)** **Pr < Po** Pr = Po
Dugan et al., 1995 (US)	2/2	1:1	10	1	Peer network for academic learning & teamwork	Obs, dur INTER	visual inspection, descriptives	**Pr < Po**
Harper et al., 2008 (US)	2/2	0:2	9	1	Peer coaches using Pivotal Response Training	Obs, N INIT gain attention Obs, N INIT play Obs, N turn-taking	visual inspection, descriptives	**Pr < Po (1 subj)** **Pr < Po (1 subj)** **Pr < Po**
Hart & Banda, 2018 (US)	1/1	0:1	6	1	Peer network	Obs, N RESP	visual inspection, descriptives	**Pr < Po**
						Obs, N INIT		Pr = Po
Hughes et al., 2013b (US)	3/3	1:2	17	2	Peers trained as partners	Obs, N P-INIT Obs, N INIT	visual inspection, descriptives	**Pr < Po** **Pr < Po**
Kamps et al., 2002 (US)	34/34	10:24	7–14	1 + 2	Peer network (trained & untrained) during play/ lunch/ recess	Obs, dur INTER Obs, reciprocity	ANOVA, visual inspection	**Pr < Po** **Pr < Po**
Pierce & Schreibman, 1997 (US)	2/2	0:2	8	1	Peer coaches using Pivotal Response Training	Obs, N INIT Obs, dur INTER maintain	visual inspection, descriptives	**Pr < Po** **Pr < Po**
Schaefer et al., 2018 (US)	3 SN/1	0:1	13	2	Peer network/coaches	Obs, dur INTER Obs, engaged level	visual inspection, descriptives	**Pr < Po** **Pr < Po**
Thiemann & Goldstein, 2004 (US)	5/5	0:5	8; 6–9	1	Peers trained as partners	Obs, N P-RESP Obs, N INIT verbal	visual inspection, descriptives	**Pr < Po** Pr = Po
Kasari et al., 2012 (US)	15/15	5:10	8; 6–11	1	Peers trained as coaches/facilitators on playgrounds (PEER)	Obs, engaged solo / joint PN, centrality PN, indegrees	HLM, ANCOVA	**C < E (d = .94 / .77)** **C < E (d = .79)** **C < E (d = .74)**
						PN, outdegrees PN, bidegree PN, rejection		C = E C = E C = E
***Peer***** + *****Researcher***								
Dean et al., 2020 (US)	32/32	4:28	15	2	Peers trained as partners/coaches + facilitator (ENGAGE)	Obs, engaged solo Obs, engaged joint	LMM, visual inspection	**Pr > Po (f**^**2**^** = .08)** **Pr < Po (f**^**2**^** = .06)**
						Obs, engaged parallel		Pr = Po (f^2^ < .01)
Frederickson et al., 2005 (UK)	14 SN/1	NA *3:11	7–11	1	Class discussion & peer network + facilitator (CIRCLE OF FRIENDS)	PN, acceptance PN, rejection	ANOVA, visual inspection, descriptives	**Pr < Po (whole class: η**^**2**^** = .30; trained: η**^**2**^** = .20; untrained: η**^**2**^** = .16)** **Pr > Po (whole class: η**^**2**^** = .40; trained: η**^**2**^** = .36; untrained: η**^**2**^** = .27)**
Haring & Breen, 1992 (US)	2 SN/1	0:1	12–13	2	Peer network + facilitator	Obs, N INTER Obs, N RESP proper Obs, N INIT unprompt PR, INTER quality	visual inspection, descriptives	**Pr < Po** **Pr < Po** **Pr < Po** **Pr < Po**
Levy & Dunsmuir, 2020 (US)	6/6	0:6	11–14	2	Lego with peers + facilitator	Obs, dur INTER Obs, N INIT Obs, N RESP	visual inspection, descriptives	**Pr < Po (Tau-U = 1.00)** **Pr < Po (Tau-U = .98)** **Pr < Po (Tau-U = .91)**
Kasari et al., 2016 (US)	80/80	19:61	8	1	Peer network, child-led activities with adult support (ENGAGE)	Obs, engaged joint	GLMM	**Pr < Po**
						PN, centrality PN, outdegree PN, indegree		Pr = Po Pr = Po Pr = Po
Sreckovic et al., 2017 (US)	3/3	0:3	15	2	Peer network + facilitator	Obs, N INIT / RESP Obs, N P-INIT / P-RESP SR, victimization	visual inspection, descriptives	**Pr < Po** **Pr < Po** **Pr > Po**
Thiemann & Goldstein, 2004 (US)	5/5	0:5	8; 6–9	1	Peers trained as partners & peer network with visual materials + facilitator	Obs, N INIT verbal Obs, N P-RESP PN, acceptance	visual inspection, descriptives	**Pr < Po** **Pr < Po** **Pr < Po**
***Peer***** + *****Staff***								
Biggs et al., 2018 (US)	4 SN/3	2:1	10	1	Peer network + paraprofessional	Obs, dur INTER verbal	visual inspection, descriptives	**Pr < Po**
Gardner et al., 2014 (US)	2/2	0:2	16	2	Peer network + staff facilitator	Obs, dur INTER Obs, engaged joint	visual inspection, descriptives	**Pr < Po** **Pr < Po**
Hochman et al., 2015 (US)	4/4	0:4	15	2	Peer network + staff facilitator	Obs, N INTER Obs, engaged joint	visual inspection, descriptives	**Pr < Po** **Pr < Po**
Huber et al., 2018 (US)	3 SN/2	0:2	15	2	Peer network + paraprofessional	Obs, N RESP Obs, reciprocity	visual inspection, descriptives	**Pr < Po** **Pr < Po**
						Obs, N INIT		Pr = Po
Hughes et al., 2011 (US)	5 SN/1	1:0	16	2	Communication books + peer partners + staff facilitator	Obs, dur INTER Obs, N INIT / RESP Obs, N P-INIT / P-RESP	visual inspection, descriptives	**Pr < Po** **Pr < Po** **Pr < Po**
Hughes et al., 2013a (US)	6/6	3:3	16–18	2	Communication books + peer coaches + staff facilitator	Obs, dur INTER Obs, N INIT / RESP Obs, N P-INIT / P-RESP	visual inspection, descriptives	**Pr < Po** **Pr < Po** **Pr < Po**
Kamps et al., 2014 (US)	2/2	0:2	6–7	1	Peer network + staff facilitator	Obs, N INIT Obs, N RESP	visual inspection, descriptives	**Pr < Po** (Tau = .66) **Pr < Po** (Tau = .74)
Mason et al., 2014 (US)	3/3	0:3	7	1	Peer network + staff facilitator	Obs, N INIT verbal	visual inspection, descriptives	**Pr < Po (**Tau = .99)
Rodríguez-Medina et al., 2016 (ES)	1/1	0:1	8	1	Recess challenges with all peers + staff facilitator & peer interview	Obs, N INIT Obs, N RESP Obs, dur INTER Obs, dur alone PN, outdegree	visual inspection, descriptives, Wilcoxon test	**Pr < Po (NAP = 66%)** **Pr < Po (NAP = 76%)** **Pr < Po** **Pr > Po** **Pr < Po (r = .78)**
						PN, acceptance		Pr = Po
Asmus et al., 2017 (US)	95 SN/45	NA	9-12th grade	2	Peer network + staff facilitator	TR, N social contacts TR, N new friends	HLM	**C < E (d = 1.39)** **C < E (d = 1.39)**
						PR, N social contacts PR, N new friends		C = E (d = .25) C = E (d = .28)
Brock et al., 2018 (US)	11/11	1:10	11	1 + 2	Peer coaches + staff facilitators using Pivotal Response Training	Obs, dur INTER Obs, N INIT Obs, N P-INIT	LMM	**C < E (d = 1.13)** **C < E (d = 1.01)** **C < E (d = .89)**
Carter et al., 2016 (US)	99 SN/42	NA	9-12th grade	2	Peer network + staff facilitator	Obs, N INTER Obs, N INIT / RESP Obs, N P-INIT / P-RESP TR, friends	HLM	**C < E (d = .42)** **C < E (d = .34)** **C < E (d = .50)** **C < E (d = 1.02)**
**Staff Level**								
Feldman & Matos, 2013 (US)	3/3	0:3	5–9	1	Paraprofessional training using Pivotal Response Training	Obs, reciprocity	visual inspection, descriptives	**Pr < Po**
Kim et al., 2017 (US)	3/3	NA	8	1	Paraprofessional training	Obs, engaged joint Obs, N INIT	visual inspection, descriptives	**Pr < Po** **Pr < Po**
Locke et al., 2019 (US)	31/31	4:27	9	1	Staff training: identify needs/facilitate interaction on playground (REMAKING RECESS)	Obs, engaged solo Obs, engaged joint PN, centrality PN, indegree	LMM, visual inspection	**Pr > Po (f = .80)** **Pr < Po (f = .80)** **Pr < Po (f = .37)** Pr = Po (f = .32)
Robinson, 2011 (US)	2/2	0:2	7–8	1	Paraprofessional training using Pivotal Response Training	Obs, dur INTER verbal	visual inspection, descriptives	**Pr < Po**
Kretzmann et al., 2015 (US)	24/24	8:16	8	1	Paraprofessional training (REMAKING RECESS)	Obs, engaged joint	LMM	**C < E (d = 1.27)**
Morgan et al., 2018 (US)	197/197 *117 special classes	37:160	7	1 + K	Staff training + continued coaching (CLASSROOM SCERTS)	Obs, engaged joint (connectedness, directed communication, generative language)	LMM	**C < E (d = .34)**
Shih et al., 2019 (US)	80/80	7:73	8	1	Staff training (REMAKING RECESS)	Obs, engaged solo PN, centrality PN, outdegree Obs, engaged joint PN, indegree	GLMM with truncated Gaussian	**C > E (f = .23)** **C < E (f = .13)** **C < E (f = .26)** C = E (f = .12) C = E (f = .13)
**Physical Environment Level**								
Baker et al., 1998 (US)	3/3	2:1	5–9	1	Make interest into playground games	Obs, engaged joint	visual inspection, descriptives	**Pr < Po**
Koegel et al., 2012 (US)	3/3	0:3	11–14	2	Social clubs formed around the interest	Obs, dur INTER Obs, N INIT	visual inspection, descriptives	**Pr < Po** Pr = Po
Koegel et al., 2013 (US)	7/7	1:6	14–16	2	Social clubs formed around the interest	Obs, engaged joint Obs, N INIT	visual inspection, descriptives	**Pr < Po** **Pr < Po**
Vincent et al., 2018 (US)	7/7 *1 kindergartener	2:5 *2:4	7	1 + K	Providing structured activities to all peers on playground (FRIEND)	Obs, engaged joint Obs, N INIT	visual inspection, descriptives	**Pr < Po** **Pr < Po**
***All 4 Levels***								
Chung & Douglas, 2015 (US)	3/3	0:3	11	1	Change seat, speech generating device, peer partner, paraprofessional facilitator	Obs, reciprocity Obs, N INIT Obs, N P-INIT	visual inspection, descriptives	**Pr < Po** **Pr < Po** **Pr < Po**

*Note*: Setting 1 = primary schools; Setting 2 = secondary schools; K = kindergarten; Obs = observations; PN = peer nomination/rating; TR = teacher reports; PR = parent reports; SR = self-reports; INIT = initiations by the focus child; RESP = responses by the focus child; P-INIT = initiations by peers; P-RESP = responses by peers; INTER = interactions; Indegree = nominations given by peers; Outdegree: nominations given by the focus child; Bidegree: reciprocated nominations; N = number/frequency; Dur = duration; Pos = positive; Neg = negative; AN(C)OVA = analysis of (co)variance; (G)LMM = (generalized) linear mixed model; HLM = hierarchical linear model; Pr = Pre-test in studies with a single-subject design; Po = Post-tests in studies with a single-subject design; C = the control group in studies with a group design; E = the experimental group in studies with a group design; NA = information not available; NAP = non-overlap of all pairs; PND = percentage of non-overlapping data; PDO = pairwise data overlap; IRD = improvement rate difference; The names of the intervention programs, when available, are shown in capital letters.

**Table 5:** Methodological quality ratings of the reviewed studies (group design)

Study	Primary indicators						Secondary indicators								Quality
	PC	IV	DV	CC	Link to RQ	Stats tests	RA	Attrition	IOA	Fidelity	Blind raters	Effect size	G/M	Social validity	
Asmus et al., 2017 (US)	Y	Y	Y	Y	Y	Y	N	N	N	Y	N	Y	Y	Y	Strong
Brock et al., 2018 (US)	Y	Y	Y	N	Y	Y	Y	Y	Y	Y	N	Y	N	Y	Adequate
Carter et al., 2016 (US)	N	Y	Y	Y	Y	Y	Y	Y	Y	Y	N	Y	Y	Y	Adequate
Golzari et al., 2015 (IR)	N	Y	Y	Y	Y	Y	Y	N	Y	N	N	Y	Y	Y	Adequate
Kasari et al., 2012 (US)	Y	Y	Y	Y	Y	Y	Y	Y	Y	Y	Y	Y	Y	N	Strong
Kretzmann et al., 2015 (US)	Y	Y	Y	N	Y	Y	Y	Y	Y	Y	Y	Y	Y	Y	Adequate
Lopata et al., 2018 (US)	Y	Y	N	N	Y	Y	Y	Y	Y	Y	Y	Y	N	N	Adequate
Morgan et al., 2018 (US)	N	Y	N	N	Y	Y	Y	Y	Y	Y	Y	Y	N	Y	Adequate
Shih et al., 2019 (US)	Y	Y	Y	Y	Y	Y	Y	Y	Y	Y	Y	Y	Y	Y	Strong

*Note*: Y = the study meets the criterion; N = the study does not meet the criterion or does not provide relevant information; PC = *participant characteristics* (i.e., age, gender, diagnoses) were provided, and if applicable, interventionist information was included; IV = *independent variables*, i.e., information about the intervention, were provided with replicable precision; DV = *dependent variables* were presented with replicable precision, linked to the intervention, and collected at suitable times; CC: *comparison condition* was defined with replicable precision, at least including any other interventions received by the participants; Link to RQ = *link between research question and analysis* was clearly made with correct units of measure; Stats tests = *use of statistical tests* was proper with sufficient power and N ≥ 10; RA = *random assignment* was used to assign the groups; Attrition = *attribution* was comparable between conditions (not different for > 25%) and < 30% for the final measure; IOA = *inter-observer/rater agreement* was collected and reported ≥ .80 and κ ≥ .60, and if applicable, psychometric properties of standardized tests were reported (agreement ≥ .70, κ ≥ .40); Fidelity = *fidelity* (procedural or treatment) was continuously assessed, and with a measurement statistics ≥ .80 if applicable; Blind raters = *Blind raters* to the condition of the participants; Effect size = *effect sizes* were reported for at least 75% of the outcome measures; G/M = *generalization and/or maintenance* were assessed; Social validity = *social validity* is considered confirmed if four of the seven criteria in the study of Reichow et al. (2008) were met

**Table 6:** Methodological quality ratings of the reviewed studies (single subject design)

Study	Primary indicators						Secondary indicators						Quality
	PC	IV	DV	Baseline condition	Visual analysis	EC	IOA	Kappa	Fidelity	Blind raters	G/M	Social validity	
Baker et al., 1998 (US)	Y	Y	Y	Y	Y	Y	N	N	N	N	Y	Y	Adequate
Biggs et al., 2018 (US)	Y	Y	Y	Y	Y	Y	Y	N	Y	N	Y	Y	Strong
Block et al., 2015 (US)	Y	Y	Y	Y	Y	Y	Y	N	Y	N	Y	Y	Strong
Bock, 2007 (US)	Y	Y	Y	Y	Y	Y	Y	N	Y	Y	Y	Y	Strong
Brain & Mirenda, 2019 (CA)	Y	Y	Y	Y	Y	Y	Y	N	N	Y	Y	Y	Strong
Carter et al., 2017 (US)	Y	Y	N	Y	Y	Y	Y	N	Y	N	N	Y	Adequate
Chung & Douglas, 2015 (US)	Y	Y	Y	Y	Y	Y	Y	N	Y	N	N	Y	Strong
Dean et al., 2020 (US)	Y	Y	N	N	Y	Y	Y	N	Y	Y	Y	N	Adequate
Delano & Snell, 2006 (US)	Y	Y	Y	Y	Y	Y	Y	Y	Y	N	Y	N	Strong
Dugan et al., 1995 (US)	Y	Y	N	Y	Y	Y	Y	N	N	N	N	Y	Adequate
Feldman & Matos, 2013 (US)	Y	Y	Y	Y	Y	Y	Y	Y	Y	N	Y	Y	Strong
Feng et al., 2008 (TW)	Y	Y	Y	Y	Y	Y	Y	N	Y	N	Y	Y	Strong
Frederickson et al., 2005 (UK)	N	Y	Y	Y	Y	Y	N	N	N	N	Y	Y	Adequate
Gardner et al., 2014 (US)	Y	Y	N	Y	Y	Y	Y	N	Y	N	N	Y	Adequate
Hanley-Hochdorfer e al., 2010 (US)	N	Y	Y	Y	Y	Y	Y	N	Y	N	Y	N	Adequate
Haring & Breen, 1992 (US)	Y	Y	Y	Y	Y	Y	Y	N	N	N	Y	N	Adequate
Harper et al., 2008 (US)	Y	Y	N	Y	Y	Y	Y	N	Y	N	Y	Y	Adequate
Hart & Banda, 2018 (US)	Y	Y	Y	Y	Y	Y	N	N	Y	N	Y	Y	Strong
Hartzell et al., 2015 (US)	Y	Y	Y	Y	Y	Y	Y	N	Y	N	Y	Y	Strong
Hochman et al., 2015 (US)	Y	Y	Y	Y	Y	Y	Y	N	Y	N	N	Y	Strong
Huber et al., 2018 (US)	Y	Y	N	Y	Y	Y	Y	N	Y	N	N	Y	Adequate
Hughes et al., 2011 (US)	Y	Y	Y	Y	Y	Y	Y	N	Y	N	Y	N	Strong
Hughes et al., 2013a (US)	Y	Y	Y	Y	Y	Y	Y	N	Y	N	Y	Y	Strong
Hughes et al., 2013b (US)	Y	Y	N	Y	Y	Y	Y	N	Y	N	Y	Y	Adequate
Kamps et al., 2002 (US)	N	Y	Y	N	Y	Y	Y	N	N	N	Y	Y	Adequate
Kamps et al., 2014 (US)	Y	Y	N	Y	Y	Y	N	N	Y	N	Y	Y	Adequate
Kasari et al., 2016 (US)	Y	Y	Y	N	N	Y	Y	N	Y	Y	Y	N	Adequate
Kim et al., 2017 (US)	N	Y	Y	Y	Y	Y	Y	Y	Y	N	Y	Y	Adequate
Koegel et al., 2012 (US)	Y	Y	N	Y	Y	Y	Y	N	N	N	N	Y	Adequate
Koegel et al., 2013 (US)	Y	Y	Y	N	Y	Y	Y	N	N	N	Y	Y	Adequate
Laushey et al., 2009 (US)	N	Y	Y	Y	Y	Y	Y	N	Y	N	Y	Y	Adequate
Levy & Dunsmuir, 2020 (UK)	Y	Y	Y	Y	Y	Y	Y	Y	Y	N	Y	Y	Strong
Locke et al., 2019 (US)	Y	Y	Y	N	Y	Y	Y	Y	Y	Y	Y	N	Adequate
Mason et al., 2014 (US)	Y	Y	N	Y	Y	Y	Y	N	Y	N	N	Y	Adequate
Pirece & Schreibman, 1997 (US)	N	Y	Y	Y	Y	Y	Y	N	N	N	Y	N	Adequate
Radley et al., 2014 (US)	Y	Y	N	Y	Y	Y	Y	Y	Y	N	N	Y	Adequate
Radley et al., 2017 (US)	Y	Y	Y	Y	Y	Y	Y	N	Y	N	Y	Y	Strong
Reutebuch et al., 2015 (US)	Y	Y	Y	Y	Y	Y	Y	N	Y	N	Y	N	Strong
Robinson, 2011 (US)	Y	Y	Y	N	Y	Y	Y	Y	Y	N	Y	Y	Adequate
Rodríguez-Medina et al., 2016 (US)	Y	Y	Y	Y	Y	Y	N	N	N	N	Y	Y	Adequate
Rosenberg et al., 2015 (US)	N	Y	N	Y	Y	Y	Y	N	N	N	Y	Y	Adequate
Sabey et al., 2020 (US)	N	Y	Y	Y	Y	N	N	N	Y	N	N	Y	Adequate
Sansosti & Powell-Smith, 2006 (US)	Y	Y	Y	Y	Y	Y	Y	N	N	N	Y	N	Adequate
Schaefer et al., 2018 (US)	Y	N	N	Y	Y	Y	Y	N	Y	N	Y	Y	Adequate
Sreckovic et al., 2017 (US)	Y	Y	Y	Y	Y	Y	Y	N	Y	N	Y	Y	Strong
Thiemann & Goldstein, 2004 (US)	Y	Y	N	Y	Y	Y	Y	N	Y	N	Y	N	Adequate
Vincent et al., 2018 (US)	Y	Y	N	Y	Y	Y	Y	N	Y	N	N	Y	Adequate

*Note*: Y = the study meets the criterion; N = the study does not meet the criterion or does not provide relevant information; PC = *participant characteristics* (i.e., age, gender, diagnoses) were provided, and if applicable, interventionist information was included; IV = *independent variables*, i.e., information about the intervention, were provided with replicable precision; DV = *dependent variables* were presented with replicable precision, linked to the intervention, and collected at suitable times; Baseline condition = *baseline condition* included ≥ 3 measurement points, was stable according to visual analysis, showed no trend, was described with replicable precision; Visual analysis = *visual analysis* was provided for all relevant data for each participant; EC = *experimental control* was present, e.g., ≥ 3 occasions of the intervention, at three different points in time, manipulation of DV/IV similar in all instances of replication; IOA = *inter-observer/rater agreement* was collected for ≥ 20% of sessions with an agreement ≥ .80; Kappa = *Kappa* was computed for ≥ 20% of sessions with κ ≥ .60; Fidelity = *fidelity* (procedural or treatment) was continuously assessed, and with a measurement statistics ≥ .80 if applicable; Blind raters = *Blind raters* to the condition of the participants; G/M = *generalization and/or maintenance* were assessed; Social validity = *social validity* is considered confirmed if four of the seven criteria in the study of Reichow et al. (2008) were met

## Declarations

### Registration

The protocol of this systematic review study was registered at Open Science Platform (https://osf.io/8u5rb).

## References

[CR1] Ackerman, K. B., Spriggs, A. D., & Rhodes, A. L. (2021). Peer mediators’ use of prompting to increase social communication in students with disabilities. *Communication Disorders Quarterly,**43*(1), 42–50. 10.1177/1525740120936999

[CR2] Allen, K. A., Slaten, C. D., Arslan, G., Roffey, S., Craig, H., & Vella-Brodrick, D. A. (2021). School belonging: The importance of student and teacher relationships. In M. L. Kern & M. L. Wehmeyer (Eds.), *The palgrave handbook of positive education. *Palgrave Macmillan. 10.1007/978-3-030-64537-3_21

[CR3] Andersen, H. B., Christiansen, L. B., Pawlowski, C. S., & Schipperijn, J. (2019). What we build makes a difference–Mapping activating schoolyard features after renewal using GIS, GPS and accelerometers. *Landscape and Urban Planning,**191*, 103617. 10.1016/j.landurbplan.2019.103617

[CR4] Asmus, J. M., Carter, E. W., Moss, C. K., Biggs, E. E., Bolt, D. M., Born, T. L., Bottema-Beutel, K., Brock, M. E., Cattey, G. N., Cooney, M., Fesperman, E. S., Hochman, J. M., Huber, H. B., Lequia, J. L., Lyons, G. L., Vincent, L. B., & Weir, K. (2017). Efficacy and social validity of peer network interventions for high school students with severe disabilities. *American Journal on Intellectual and Developmental Disabilities,**122*(2), 118–137. 10.1352/1944-7558-122.2.118 28257242 10.1352/1944-7558-122.2.118

[CR5] Baker, M. J., Koegel, R. L., & Koegel, L. K. (1998). Increasing the social behavior of young children with autism using their obsessive behaviors. *Journal of the Association for Persons with Severe Handicaps,**23*(4), 300–308. 10.2511/rpsd.23.4.300

[CR6] Bailey, J., & Baker, S. T. (2020). A synthesis of the quantitative literature on autistic pupils’ experience of barriers to inclusion in mainstream schools. *Journal of Research in Special Educational Needs,**20*, 291–307. 10.1111/1471-3802.12490

[CR7] Bailey, B., Zyga, O., Meeker, H., Kirk, J., & Russ, S. W. (2021). A comparison of curricula: Examining the efficacy of a school-based musical theater intervention. *Journal of Intellectual Disabilities,**25*(3), 370–386. 10.1177/1744629519883159 31750754 10.1177/1744629519883159

[CR8] Bambara, L. M., Cole, C. L., Kunsch, C., Tsai, S. C., & Ayad, E. (2016). A peer-mediated intervention to improve the conversational skills of high school students with autism spectrum disorder. *Research in Autism Spectrum Disorders,**27*, 29–43. 10.1016/j.rasd.2016.03.003

[CR9] Biggs, E. E., Carter, E. W., Bumble, J. L., Barnes, K., & Mazur, E. L. (2018). Enhancing peer network interventions for students with complex communication needs. *Exceptional Children,**85*(1), 66–85. 10.1177/0014402918792899

[CR10] Block, H. M., Radley, K. C., Jenson, W. R., Clark, E., & O’Neill, R. E. (2015). Effects of a multimedia social skills program in increasing social responses and initiations of children with Autism Spectrum Disorder. *International Journal of School and Educational Psychology,**3*(1), 16–24. 10.1080/21683603.2014.923355

[CR11] Bock, M. A. (2007). The Impact of social-behavioral learning strategy training on the social interaction skills of four students with Asperger Syndrome. *Focus on Autism and Other Developmental Disabilities,**22*(2), 88–95. 10.1177/10883576070220020901

[CR12] Boyd, B. A., Watson, L. R., Reszka, S. S., Sideris, J., Alessandri, M., Baranek, G. T., Crais, E. R., Donaldson, A. L., Gutierrez, A., Johnson, L. D., & Belardi, K. (2018). Efficacy of the ASAP intervention for preschoolers with ASD: A cluster randomized controlled trial. *Journal of Autism and Developmental Disorders,**48*(9), 3144–3162. 10.1007/s10803-018-3584-z 29691794 10.1007/s10803-018-3584-z

[CR13] Brain, T., & Mirenda, P. (2019). Effectiveness of a low-intensity peer-mediated intervention for middle school students with autism spectrum disorder. *Research in Autism Spectrum Disorders,**62*, 26–38. 10.1016/j.rasd.2019.02.003

[CR14] Brewster, S., & Coleyshaw, L. (2011). Participation or exclusion? Perspectives of pupils with autistic spectrum disorders on their participation in leisure activities. *British Journal of Learning Disabilities,**39*(4), 284–291. 10.1111/j.1468-3156.2010.00665.x

[CR15] Brock, M. E., Dueker, S. A., & Barczak, M. A. (2018). Brief Report: Improving social outcomes for students with autism at recess through peer-mediated pivotal response training. *Journal of Autism and Developmental Disorders,**48*(6), 2224–2230. 10.1007/s10803-017-3435-3 29255960 10.1007/s10803-017-3435-3

[CR16] Buchanan, D., Hargreaves, E., & Quick, L. (2022). Schools closed during the pandemic: revelations about the well-being of ‘lower-attaining’ primary-school children. *Education 3-13*, *51*(7), 1077–1090. 10.1080/03004279.2022.2043405

[CR17] Camargo, S. P. H., Rispoli, M., Ganz, J. B., Hong, E. R., Davis, H., & Mason, R. A. (2014). A Review of the quality of behaviorally-based intervention research to improve social interaction skills of children with ASD in inclusive settings. *Journal of Autism and Developmental Disorders,**44*(9), 2096–2116. 10.1007/s10803-014-2060-7 24781498 10.1007/s10803-014-2060-7

[CR18] Carter, E. W., Asmus, J., Moss, C. K., Biggs, E. E., Bolt, D. M., Born, T. L., Brock, M. E., Cattey, G. N., Chen, R., Cooney, M., Fesperman, E., Hochman, J. M., Huber, H. B., Lequia, J. L., Lyons, G., Moyseenko, K. A., Riesch, L. M., Shalev, R. A., Vincent, L. B., & Weir, K. (2016). Randomized evaluation of peer support arrangements to support the inclusion of high school students with severe disabilities. *Exceptional Children,**82*(2), 209–233. 10.1177/0014402915598780

[CR19] Carter, E. W., Gustafson, J. R., Sreckovic, M. A., Dykstra Steinbrenner, J. R., Pierce, N. P., Bord, A., Stabel, A., Rogers, S., Czerw, A., & Mullins, T. (2017). Efficacy of peer support interventions in general education classrooms for high school students with Autism Spectrum Disorder. *Remedial and Special Education,**38*(4), 207–221. 10.1177/0741932516672067

[CR20] Chung, Y. C., & Douglas, K. H. (2015). A peer interaction package for students with Autism Spectrum Disorders who use speech-generating devices. *Journal of Developmental and Physical Disabilities,**27*(6), 831–849. 10.1007/s10882-015-9461-1

[CR21] Corbett, B. A., Swain, D. M., Coke, C., Simon, D. M., Newsom, C. R., Houchins-Juarez, N., Jenson, A., Wang, L., & Song, Y. (2013). Improvement in social deficits in autism spectrum disorders using a Theatre-Based, Peer-Mediated intervention. *Autism Research,**7*(1), 4–16. 10.1002/aur.1341 24150989 10.1002/aur.1341 PMC3943749

[CR22] De Boer, A., & Pijl, S. J. (2016). The acceptance and rejection of peers with ADHD and ASD in general secondary education. *Journal of Educational Research,**109*(3), 325–332. 10.1080/00220671.2014.958812

[CR23] Dean, M., & Chang, Y.-C. (2021). A systematic review of school-based social skills interventions and observed social outcomes for students with autism spectrum disorder in inclusive settings. *Autism,**25*(7), 1828–1843. 10.1177/13623613211012886 34231405 10.1177/13623613211012886

[CR24] Dean, M., Kasari, C., Shih, W., Frankel, F., Whitney, R., Landa, R., Lord, C., Orlich, F., King, B., & Harwood, R. (2014). The peer relationships of girls with ASD at school: Comparison to boys and girls with and without ASD. *Journal of Child Psychology and Psychiatry,**55*, 1218–1225. 10.1111/jcpp.12242 25039696 10.1111/jcpp.12242 PMC4269475

[CR25] Dean, M., Williams, J., Orlich, F., & Kasari, C. (2020). Adolescents with Autism Spectrum Disorder and social skills groups at school: A randomized trial comparing intervention environment and peer composition. *School Psychology Review,**49*(1), 60–73. 10.1080/2372966X.2020.1716636 33041430 10.1080/2372966x.2020.1716636 PMC7540922

[CR26] Delano, M., & Snell, M. E. (2006). The effects of social stories on the social engagement of children with autism. *Journal of Positive Behavior Interventions,**8*(1), 29–42. 10.1177/10983007060080010501

[CR27] Dugan, E., Kamps, D., Leonard, B., Watkins, N., Rheinberger, A., & Stackhaus, J. (1995). Effects of cooperative learning groups during social studies for students with autism and fourth-grade peers. *Journal of Applied Behavior Analysis,**28*(2), 175–188. 10.1901/jaba.1995.28-175 7601803 10.1901/jaba.1995.28-175 PMC1279808

[CR28] Eichengreen, A., van Rooijen, M., van Klaveren, L.-M., Nasri, M., Tsou, Y. T., Koutamanis, A., Baratchi, M., & Rieffe, C. (2024). The impact of loose-parts-play on schoolyard social participation of children with and without disabilities: A case study. *Child: Care, Health and Development*, *50*(1), e13144. 10.1111/cch.13144 10.1111/cch.13144 37322578

[CR29] Ezzamel, N. (2016). *Peer-mediated Interventions for Pupils with ASD in Mainstream Schools; a Tool to Promote Social Inclusion*. The University of Manchester (United Kingdom).

[CR30] Feldman, E. K., & Matos, R. (2013). Training paraprofessionals to facilitate social interactions between children with autism and their typically developing peers. *Journal of Positive Behavior Interventions,**15*(3), 169–179. 10.1177/1098300712457421

[CR31] Feng, H., Lo, Y., Tsai, S., & Cartledge, G. (2008). The effects of Theory-of-Mind and social skill training on the social competence of a sixth-grade student with autism. *Journal of Positive Behavior Interventions,**10*(4), 228–242. 10.1177/1098300708319906

[CR32] Frederickson, N., Warren, L., & Turner, J. (2005). “Circle of Friends”—An exploration of impact over time. *Educational Psychology in Practice,**21*(3), 197–217. 10.1080/02667360500205883

[CR33] Gardner, K. F., Carter, E. W., Gustafson, J. R., Hochman, J. M., Harvey, M. N., Mullins, T. S., & Fan, H. (2014). Effects of peer networks on the social interactions of high school students with Autism Spectrum Disorders. *Research and Practice for Persons with Severe Disabilities,**39*(2), 100–118. 10.1177/1540796914544550

[CR34] Gray, C. A. (2010). *The New Social Story Book*. Future Horizons.

[CR35] Gray, C. A., & Garand, J. D. (1993). Social Stories: Improving responses of students with autism with accurate social information. *Focus on Autistic Behavior,**8*(1), 1–10. 10.1177/108835769300800101

[CR36] Golzari, F., Hemati Alamdarloo, G., & Moradi, S. (2015). The effect of a Social Stories intervention on the social skills of male students with Autism Spectrum Disorder. *SAGE Open*, *5*(4). 10.1177/2158244015621599

[CR37] Hanley-Hochdorfer, K., Bray, M. A., Kehle, T. J., & Elinoff, M. J. (2010). Social Stories to increase verbal initiation in children with autism and Asperger’s disorder. *School Psychology Review,**39*(3), 484–492. 10.1080/02796015.2010.12087767

[CR38] Haring, T. G., & Breen, C. G. (1992). A peer-mediated social network intervention to enhance the social integration of persons with moderate and severe disabilities. *Journal of Applied Behavior Analysis,**25*(2), 319–333. 10.1901/jaba.1992.25-319 1634425 10.1901/jaba.1992.25-319 PMC1279713

[CR39] Harper, C. B., Symon, J. B. G., & Frea, W. D. (2008). Recess is time-in: Using peers to improve social skills of children with autism. *Journal of Autism and Developmental Disorders,**38*(5), 815–826. 10.1007/s10803-007-0449-2 17874290 10.1007/s10803-007-0449-2

[CR40] Hart, S. L., & Banda, D. R. (2018). Examining the effects of peer mediation on the social skills of students with Autism Spectrum Disorder as compared to their peers. *Education and Training in Autism and Developmental Disabilities,**53*(2), 160–175. 10.2307/26495267

[CR41] Hartzell, R., Liaupsin, C., Gann, C. and Clem, S. (2015). Increasing social engagement in an inclusive environment. *Education and Training in Autism and Developmental Disabilities,**50*(3), 264–277. https://www.jstor.org/stable/24827509

[CR42] Hochman, J. M., Carter, E. W., Bottema-Beutel, K., Harvey, M. N., & Gustafson, J. R. (2015). Efficacy of peer networks to increase social connections among high school students with and without autism spectrum disorder. *Exceptional Children,**82*(1), 96–116. 10.1177/0014402915585482

[CR43] Hoffman, J. A., & Miller, E. A. (2020). Addressing the consequences of school closure due to COVID-19 on children’s physical and mental well-being. *World Medical & Health Policy,**12*, 300–310. 10.1002/wmh3.365 32904951 10.1002/wmh3.365 PMC7461306

[CR44] Huber, H. B., Carter, E. W., Lopano, S. E., & Stankiewicz, K. C. (2018). Using structural analysis to inform peer support arrangements for high school students with severe disabilities. *American Journal on Intellectual and Developmental Disabilities,**123*(2), 119–139. 10.1352/1944-7558-123.2.119 29480778 10.1352/1944-7558-123.2.119

[CR45] Hughes, C., Bernstein, R. T., Kaplan, L. M., Reilly, C. M., Brigham, N. L., Cosgriff, J. C., & Boykin, M. P. (2013a). Increasing conversational interactions between verbal high school students with autism and their peers without disabilities. *Focus on Autism and Other Developmental Disabilities,**28*(4), 241–254. 10.1177/1088357613487019

[CR46] Hughes, C., Golas, M., Cosgriff, J., Brigham, N., Edwards, C., & Cashen, K. (2011). Effects of a social skills intervention among high school students with intellectual disabilities and autism and their general education peers. *Research and Practice for Persons with Severe Disabilities,**36*(1–2), 46–61. 10.2511/rpsd.36.1-2.46

[CR47] Hughes, C., Harvey, M., Cosgriff, J., Reilly, C., Heilingoetter, J., Brigham, N., Kaplan, L., & Bernstein, R. (2013b). A peer-delivered social interaction intervention for high school students with autism. *Research and Practice for Persons with Severe Disabilities,**38*(1), 1–16. 10.2511/027494813807046999

[CR48] Kamps, D., Royer, J., Dugan, E., Kravits, T., Gonzalez-Lopez, A., García, J., Carnazzo, K., Morrison, L., & Kane, L. G. (2002). Peer training to facilitate social interaction for elementary students with autism and their peers. *Exceptional Children,**68*(2), 173–187. 10.1177/001440290206800202

[CR49] Kamps, D., Mason, R., Thiemann-Bourque, K., Feldmiller, S., Turcotte, A., & Miller, T. (2014). The use of peer networks to increase communicative acts of students with autism spectrum disorders. *Focus on Autism and Other Developmental Disabilities,**29*(4), 230–245. 10.1177/1088357614539832 26312013 10.1177/1088357614539832 PMC4547562

[CR50] Kasari, C., Dean, M., Kretzmann, M., Shih, W., Orlich, F., Whitney, R., Landa, R., Lord, C., & King, B. (2016). Children with autism spectrum disorder and social skills groups at school: A randomized trial comparing intervention approach and peer composition. *Journal of Child Psychology and Psychiatry and Allied Disciplines,**57*(2), 171–179. 10.1111/jcpp.12460 26391889 10.1111/jcpp.12460

[CR51] Kasari, C., Rotheram-Fuller, E., Locke, J., & Gulsrud, A. (2012). Making the connection: Randomized controlled trial of social skills at school for children with autism spectrum disorders. *Journal of Child Psychology and Psychiatry and Allied Disciplines,**53*(4), 431–439. 10.1111/j.1469-7610.2011.02493.x 22118062 10.1111/j.1469-7610.2011.02493.x PMC3238795

[CR52] Kim, S., Koegel, R. L., & Koegel, L. K. (2017). Training paraprofessionals to target socialization in students with ASD: Fidelity of implementation and social validity. *Journal of Positive Behavior Interventions,**19*(2), 102–114. 10.1177/1098300716669813

[CR53] Koegel, R., Kim, S., Koegel, L., & Schwartzman, B. (2013). Improving socialization for high school students with ASD by using their preferred interests. *Journal of Autism and Developmental Disorders,**43*(9), 2121–2134. 10.1007/s10803-013-1765-3 23361918 10.1007/s10803-013-1765-3 PMC3672252

[CR54] Koegel, R. L., Fredeen, R., Kim, S., Danial, J., Rubinstein, D., & Koegel, L. (2012). Using perseverative interests to improve interactions between adolescents with autism and their typical peers in school settings. *Journal of Positive Behavior Interventions,**14*(3), 133–141. 10.1177/1098300712437043 24163577 10.1177/1098300712437043 PMC3806136

[CR55] Kretzmann, M., Shih, W., & Kasari, C. (2015). Improving peer engagement of children with autism on the school playground: A randomized controlled trial. *Behavior Therapy,**46*(1), 20–28. 10.1016/j.beth.2014.03.006 25526832 10.1016/j.beth.2014.03.006

[CR56] Lang, R., Kuriakose, S., Lyons, G., Mulloy, A., Boutot, A., Britt, C., Caruthers, S., Ortega, L., O’Reilly, M. F., & Lancioni, G. E. (2011). Use of school recess time in the education and treatment of children with autism spectrum disorders: A systematic review. *Research in Autism Spectrum Disorders,**5*(4), 1296–1305. 10.1016/j.rasd.2011.02.012

[CR57] Laushey, K. M., Heflin, L. J., Shippen, M., Alberto, P. A., & Fredrick, L. (2009). Concept mastery routines to teach social skills to elementary children with high functioning autism. *Journal of Autism and Developmental Disorders,**39*(10), 1435–1448. 10.1007/s10803-009-0757-9 19472042 10.1007/s10803-009-0757-9

[CR58] Leifler, E., Carpelan, G., Zakrevska, A., Bölte, S., & Jonsson, U. (2021). Does the learning environment ‘make the grade’? A systematic review of accommodations for children on the autism spectrum in mainstream school. *Scandinavian Journal of Occupational Therapy,**28*(8), 582–597. 10.1080/11038128.2020.1832145 33078981 10.1080/11038128.2020.1832145

[CR59] Levy, J., & Dunsmuir, S. (2020). Lego therapy: Building social skills for adolescents with an autism spectrum disorder. *Educational and Child Psychology*, *37*(1), 58–83. 10.53841/bpsecp.2020.37.1.58

[CR60] Locke, J., Shih, W., Kang-Yi, C. D., Caramanico, J., Shingledecker, T., Gibson, J., Frederick, L., & Mandell, D. S. (2019). The impact of implementation support on the use of a social engagement intervention for children with autism in public schools. *Autism,**23*(4), 834–845. 10.1177/1362361318787802 29998740 10.1177/1362361318787802 PMC6312760

[CR61] Lopata, C., Thomeer, M. L., Rodgers, J. D., Donnelly, J. P., McDonald, C. A., Volker, M. A., Smith, T. H., & Wang, H. (2019). Cluster randomized trial of a school intervention for children with Autism Spectrum Disorder. *Journal of Clinical Child and Adolescent Psychology,**48*(6), 922–933. 10.1080/15374416.2018.1520121 30376652 10.1080/15374416.2018.1520121

[CR62] Martin, C. S. (2016). Exploring the impact of the design of the physical classroom environment on young children with autism spectrum disorder (ASD). *Journal of Research in Special Educational Needs,**16*(4), 280–298. 10.1111/1471-3802.12092

[CR63] Martín, J. a. G., Pérez, V., Sacristán, M., & Álvarez, E. (2005). Is grey literature essential for a better control of publication bias in psychiatry? An example from three meta-analyses of schizophrenia. *European Psychiatry,**20*(8), 550–553. 10.1016/j.eurpsy.2005.03.011 10.1016/j.eurpsy.2005.03.011 15994063

[CR64] Moher, D., Liberati, A., Tetzlaff, J., & Altman, D. G. (2010). Preferred reporting items for systematic reviews and meta-analyses: The PRISMA statement. *International Journal of Surgery,**8*(5), 336–341. 10.1016/j.ijsu.2010.02.007 20171303 10.1016/j.ijsu.2010.02.007

[CR65] Morgan, L., Hooker, J. L., Sparapani, N., Reinhardt, V. P., Schatschneider, C., & Wetherby, A. M. (2018). Cluster randomized trial of the classroom SCERTS intervention for elementary students with autism spectrum disorder. *Journal of Consulting and Clinical Psychology,**86*(7), 631–644. 10.1037/ccp0000314 29939056 10.1037/ccp0000314 PMC6457665

[CR66] Mason, R., Kamps, D., Turcotte, A., Cox, S., Feldmiller, S., & Miller, T. (2014). Peer mediation to increase communication and interaction at recess for students with autism spectrum disorders. *Research in Autism Spectrum Disorders,**8*(3), 334–344. 10.1016/j.rasd.2013.12.014 26180543 10.1016/j.rasd.2013.12.014 PMC4500175

[CR67] Mayes, S. D., Calhoun, S. L., Aggarwal, R., Baker, C., Mathapati, S., Molitoris, S., & Mayes, R. D. (2013). Unusual fears in children with autism. *Research in Autism Spectrum Disorders,**7*(1), 151–158. 10.1016/j.rasd.2012.08.002

[CR68] Nasri, M., Tsou, Y. T., Koutamanis, A., Baratchi, M., Giest, S., Reidsma, D., & Rieffe, C. (2022). A novel data-driven approach to examine children’s movements and social behaviour in schoolyard environments. *Children,**9*(8), 1177. 10.3390/children9081177 36010066 10.3390/children9081177 PMC9407003

[CR69] Palikara, O., Castro-Kemp, S., Gaona, C., & Eirinaki, V. (2021). The mediating role of school belonging in the relationship between socioemotional well-being and loneliness in primary school age children. *Australian Journal of Psychology,**73*(1), 24–34. 10.1080/00049530.2021.1882270

[CR70] Petursdottir, A. I., & Carr, J. E. (2018). Applying the taxonomy of validity threats from mainstream research design to single-case experiments in applied behavior analysis. *Behavior Analysis in Practice,**11*, 228–240. 10.1007/s40617-018-00294-6 30363794 10.1007/s40617-018-00294-6 PMC6182849

[CR71] Pierce, K., & Schreibman, L. (1997). Multiple peer use of pivotal response training to increase social behaviors of classmates with autism: Results from trained and untrained peers. *Journal of Applied Behavior Analysis,**30*(1), 157–160. 10.1901/jaba.1997.30-157 9103991 10.1901/jaba.1997.30-157 PMC1284029

[CR72] Radley, K. C., Ford, W. B., Battaglia, A. A., & McHugh, M. B. (2014). The effects of a social skills training package on social engagement of children with autism spectrum disorders in a generalized recess setting. *Focus on Autism and Other Developmental Disabilities,**29*(4), 216–229. 10.1177/1088357614525660

[CR73] Radley, K. C., McHugh, M. B., Taber, T., Battaglia, A. A., & Ford, W. B. (2017). School-based social skills training for children with Autism Spectrum Disorder. *Focus on Autism and Other Developmental Disabilities,**32*(4), 256–268. 10.1177/1088357615583470

[CR74] Reichow, B., Volkmar, F. R., & Cicchetti, D. V. (2008). Development of the evaluative method for evaluating and determining evidence-based practices in autism. *Journal of Autism and Developmental Disorders,**38*(7), 1311–1319. 10.1007/s10803-007-0517-7 18095149 10.1007/s10803-007-0517-7

[CR75] Rieffe, C., Broekhof, E., Eichengreen, A., Kouwenberg, M., Veiga, G., Da Silva, B. M. S., Van der Laan, A., & Frijns, J. H. M. (2018). Friendship and emotion control in pre-adolescents with or without hearing loss. *Journal of Deaf Studies and Deaf Education,**23*, 209–218. 10.1093/deafed/eny012 29733358 10.1093/deafed/eny012

[CR76] Rieffe, C., Kamp, S., Pentinga, J., Becker, M., van Klaveren, L., & Blijd-Hoogewys, E. (2021). Sociale inclusie en ASS op middelbare scholen, wat is er nodig? *Wetenschappelijk Tijdschrift Autisme,**20*(3), 51–59.

[CR77] Reutebuch, C. K., el Zein, F., Kim, M. K., Weinberg, A. N., & Vaughn, S. (2015). Investigating a reading comprehension intervention for high school students with autism spectrum disorder: A pilot study. *Research in Autism Spectrum Disorders,**9*, 96–111. 10.1016/j.rasd.2014.10.002

[CR78] Robinson, S. E. (2011). Teaching paraprofessionals of students with autism to implement pivotal response treatment in inclusive school settings using a brief video feedback training package. *Focus on Autism and Other Developmental Disabilities,**26*(2), 105–118. 10.1177/1088357611407063

[CR79] Rodríguez-Medina, J., Martín-Antón, L. J., Carbonero, M. A., & Ovejero, A. (2016). Peer-mediated intervention for the development of social interaction skills in high-functioning autism spectrum disorder: A pilot study. *Frontiers in Psychology*, *7*. 10.3389/fpsyg.2016.01986 10.3389/fpsyg.2016.01986 PMC5179565 28066303

[CR80] Rosenberg, N., Congdon, M., Schwartz, I., & Kamps, D. (2015). Use of say-do correspondence training to increase generalizationof social interaction skills at recess for children with autism spectrum disorder. *Education and Training in Autism and Developmental Disabilities*, *50*(2), 213–222. http://www.jstor.org/stable/24827536

[CR81] Sabey, C., Ross, S., & Goodman, J. (2020). Beyond topography: Addressing the functional impact of social skills training for students with autism. *Educational Psychology in Practice,**36*(2), 133–148. 10.1080/02667363.2019.1703650

[CR82] Sansosti, F. J., & Powell-Smith, K. A. (2006). Using Social Stories to improve the social behavior of children with Asperger Syndrome. *Journal of Positive Behavior Interventions,**8*(1), 43–57. 10.1177/10983007060080010601

[CR83] Schaefer, J. M., Cannella-Malone, H., & Brock, M. E. (2018). Effects of peer support arrangements across instructional formats and environments for students with severe disabilities. *Remedial and Special Education,**39*(1), 3–14. 10.1177/0741932517727865

[CR84] Schmucker, C., Blümle, A., Schell, L. K., Schwarzer, G., Oeller, P., Cabrera, L. Y., Von Elm, E., Briel, M., & Meerpohl, J. J. (2017). Systematic review finds that study data not published in full text articles have unclear impact on meta-analyses results in medical research. *PLOS ONE,**12*(4), e0176210. 10.1371/journal.pone.0176210 10.1371/journal.pone.0176210 PMC5404772 28441452

[CR85] Shih, W., Dean, M., Kretzmann, M., Locke, J., Senturk, D., Mandell, D. S., Smith, T., & Kasari, C. (2019). Remaking Recess intervention for improving peer interactions at school for children with Autism Spectrum Disorder: Multisite randomized trial. *School Psychology Review,**48*(2), 133–144. 10.17105/SPR-2017-0113.V48-2

[CR86] Sreckovic, M. A., Hume, K., & Able, H. (2017). Examining the efficacy of peer network interventions on the social interactions of high school students with Autism Spectrum Disorder. *Journal of Autism and Developmental Disorders,**47*(8), 2556–2574. 10.1007/s10803-017-3171-8 28567546 10.1007/s10803-017-3171-8

[CR87] Sutton, B. M., Webster, A. A., & Westerveld, M. F. (2019). A systematic review of school-based interventions targeting social communication behaviors for students with autism. *Autism,**23*(2), 274–286. 10.1177/1362361317753564 29382208 10.1177/1362361317753564

[CR88] Tincani, M., & Travers, J. (2019). Replication research, publication bias, and applied behavior analysis. *Perspectives on Behavior Science,**42*(1), 59–75. 10.1007/s40614-019-00191-5 31976421 10.1007/s40614-019-00191-5 PMC6701502

[CR89] Thiemann, K. S., & Goldstein, H. (2004). Effects of peer training and written text cueing on social communication of school-age children with pervasive developmental disorder. *Journal of Speech, Language, and Hearing Research,**47*(1), 126–144. 10.1044/1092-4388(2004/012) 15072534 10.1044/1092-4388(2004/012)

[CR90] Tian, L., Tian, Q., & Huebner, E. S. (2016). School-related social support and adolescents’ school-related subjective well-being: The mediating role of basic psychological needs satisfaction at school. *Social Indicators Research,**128*(1), 105–129. 10.1007/s11205-015-1021-7

[CR91] Turnock, A., Langley, K., & Jones, C. R. G. (2022). Understanding stigma in autism: A narrative review and theoretical model. *Autism in Adulthood,**4*(1), 76–91. 10.1089/aut.2021.0005 36605561 10.1089/aut.2021.0005 PMC8992913

[CR92] Veiga, G., de Leng, W., Cachucho, R., Ketelaar, L., Kok, J. N., Knobbe, A., Neto, C., & Rieffe, C. (2017). Social competence at the playground: Preschoolers during recess. *Infant and Child Development,**26*, e1957. 10.1002/icd.1957

[CR93] Vincent, L. B., Openden, D., Gentry, J. A., Long, L. A., & Matthews, N. L. (2018). Promoting social learning at recess for children with ASD and related social challenges. *Behavior Analysis in Practice,**11*(1), 19–33. 10.1007/s40617-017-0178-8 29556445 10.1007/s40617-017-0178-8 PMC5843568

[CR94] Watkins, L., O’Reilly, M., Kuhn, M., Gevarter, C., Lancioni, G. E., Sigafoos, J., & Lang, R. (2015). A review of peer-mediated social interaction interventions for students with autism in inclusive settings. *Journal of Autism and Developmental Disorders,**45*(4), 1070–1083. 10.1007/s10803-014-2264-x 25272953 10.1007/s10803-014-2264-x

[CR95] Watkins, L., Ledbetter-Cho, K., O’Reilly, M., Barnard-Brak, L., & Garcia-Grau, P. (2019). Interventions for students with autism in inclusive settings: A best-evidence synthesis and meta-analysis. *Psychological Bulletin,**145*(5), 490–507. 10.1037/bul0000190 30869925 10.1037/bul0000190

[CR96] Weaver, L. A., Bingham, E., Luo, K., Juárez, A. P., & Taylor, J. L. (2021). What do we really mean by “inclusion?”: The importance of terminology when discussing approaches to community engagement. *Autism,**25*(8), 2149–2151. 10.1177/13623613211046688 34519551 10.1177/13623613211046688

[CR97] Whalon, K., Conroy, M. A., Martínez, J. R., & Werch, B. L. (2015). School-based peer-related social competence interventions for children with Autism Spectrum Disorder: A meta-analysis and descriptive review of single case research design studies. *Journal of Autism and Developmental Disorders,**45*(6), 1513–1531. 10.1007/s10803-015-2373-1 25641004 10.1007/s10803-015-2373-1

[CR98] Williams, E. I., Gleeson, K., & Jones, B. E. (2019). How pupils on the autism spectrum make sense of themselves in the context of their experiences in a mainstream school setting: A qualitative metasynthesis. *Autism,**23*(1), 8–28. 10.1177/1362361317723836 29139322 10.1177/1362361317723836

[CR99] Yuill, N., Strieth, S., Roake, C., Aspden, R., & Todd, B. (2007). Brief report: Designing a playground for children with autistic spectrum disorders––effects on playful peer interactions. *Journal of Autism and Developmental Disorders,**37*(6), 1192–1196. 10.1007/s10803-006-0241-8 17063401 10.1007/s10803-006-0241-8

